# Optimizing Deep Learning Models with Improved BWO for TEC Prediction

**DOI:** 10.3390/biomimetics9090575

**Published:** 2024-09-22

**Authors:** Yi Chen, Haijun Liu, Weifeng Shan, Yuan Yao, Lili Xing, Haoran Wang, Kunpeng Zhang

**Affiliations:** 1 Institute of Intelligent Emergency Information Processing, Institute of Disaster Prevention, Langfang 065201, China; 22661354@st.cidp.edu.cn (Y.C.); shanweifeng@cidp.edu.cn (W.S.); xinglili@cidp.edu.cn (L.X.); 22661333@st.cidp.edu.cn (H.W.); 2 Institute of Mineral Resources Research, China Metallurgical Geology Bureau, Beijing 101300, China; yaoyuan@cmgb.cn; 3 College of Computer Science and Technology, Jilin University, Changchun 130012, China; zhangkp18@mails.jlu.edu.cn

**Keywords:** TEC prediction, hyperparameter optimization, beluga whale optimization, swarm intelligence, deep learning model optimization

## Abstract

The prediction of total ionospheric electron content (TEC) is of great significance for space weather monitoring and wireless communication. Recently, deep learning models have become increasingly popular in TEC prediction. However, these deep learning models usually contain a large number of hyperparameters. Finding the optimal hyperparameters (also known as hyperparameter optimization) is currently a great challenge, directly affecting the predictive performance of the deep learning models. The Beluga Whale Optimization (BWO) algorithm is a swarm intelligence optimization algorithm that can be used to optimize hyperparameters of deep learning models. However, it is easy to fall into local minima. This paper analyzed the drawbacks of BWO and proposed an improved BWO algorithm, named FAMBWO (Firefly Assisted Multi-strategy Beluga Whale Optimization). Our proposed FAMBWO was compared with 11 state-of-the-art swarm intelligence optimization algorithms on 30 benchmark functions, and the results showed that our improved algorithm had faster convergence speed and better solutions on almost all benchmark functions. Then we proposed an automated machine learning framework FAMBWO-MA-BiLSTM for TEC prediction, where MA-BiLSTM is for TEC prediction and FAMBWO for hyperparameters optimization. We compared it with grid search, random search, Bayesian optimization algorithm and beluga whale optimization algorithm. Results showed that the MA-BiLSTM model optimized by FAMBWO is significantly better than the MA-BiLSTM model optimized by grid search, random search, Bayesian optimization algorithm, and BWO.

## 1. Introduction

The prediction of total electron content (TEC) in the ionosphere is of great significance for positioning and navigation, space weather monitoring, and wireless communication [1,2,3]. However, many factors affect the ionospheric TEC, such as local time, latitude, longitude, season, solar cycle, solar activity, and geomagnetic activity, and it is very difficult to establish physical prediction models for ionospheric TEC [4]. Since the establishment of the International GNSS Service (IGS) in 1998, many analysis centers, such as the European Orbital Determination Center (CODE), the European Space Agency (ESA), the Jet Propulsion Laboratory (JPL) of the United States, and the University of Technology of Catalonia (UPC), have been providing users with a Global Ionospheric Map (GIM), which provides rich data support for ionospheric TEC prediction using deep learning models. In recent years, research has shown that deep learning models outperform empirical and statistical models in ionospheric TEC prediction [5,6]. Deep learning models have become the mainstream ionospheric TEC prediction technology [7,8]. These deep learning models often contain a large number of hyperparameters. Nils’ research has shown that deep learning models can have over 12 common hyperparameters [9], including learning rate, batch size, number of hidden layer nodes, convolutional kernel size, etc. These hyperparameters are used to train deep learning models. They cannot be estimated by the models themselves [10,11,12,13]. Finding the optimal hyperparameter combination for deep learning models, also known as hyperparameter optimization, directly affects the performance of the models. Researchers have shown that finding the best hyperparameters is the main challenge in training deep learning models and is even more important than selecting deep learning models [11,14].

When optimizing hyperparameters, the first step is to define a search space that includes the hyperparameters to be optimized and the search range corresponding to them. Then, a heuristic algorithm needs to be defined to search for the best solution in the search space. Due to the wide range for each hyperparameter, there are a large number of hyperparameter combinations in the search space. Hyperparameter optimization requires evaluating all possible hyperparameter combinations to find the optimal one. Therefore, the cost of hyperparameter optimization is very expensive [11,15].

Recently, swarm intelligence optimization algorithms have been proven to be effective in hyperparameter automatic optimization [16]. The inspiration for swarm intelligence optimization algorithms comes from the swarm intelligence behavior of various animals or humans. They are simple and flexible, so many researchers use them to quickly and accurately find the global optimal solution in complex optimization problems [17,18,19]. At present, common swarm intelligence optimization algorithms include Particle Swarm Optimization (PSO) [20], Moth Flame Optimization (MFO) [21], Sine Cosine Algorithm (SCA) [22], Salp Swarm Algorithm (SSA) [23], Whale Optimization Algorithm (WOA) [24], Seagull Optimization Algorithm (SOA) [25], Grey Wolf Optimization (GWO) algorithm [26], Dung Beetle Optimization (DBO) algorithm [27], and War Strategy Optimization algorithm (WSO) [28], etc. The Beluga Whale Optimization (BWO) algorithm is a swarm intelligence optimization algorithm proposed in recent years to simulate the collaborative behavior of the beluga whale population [29]. Its performance is proved to be superior to PSO, GWO, HHO, MFO, WOA, SOA, SSA, etc. However, the original BWO algorithm has two drawbacks: (1) the initial population lacked diversity, which limited the algorithm’s search ability; (2) The exploration phase and exploitation phase are imbalanced, making it easy to fall into local optima during optimization. In order to solve the above two problems, we improved the BWO algorithm and proposed the FAMBWO algorithm. Finally, we propose a deep learning model for TEC prediction and apply our improved FAMBWO algorithm to optimize the hyperparameters of the deep learning model. The contributions of this paper are as follows:

- To improve the diversity of the initial population, we used Cat Chaotic Mapping (CCM) to initialize the initial population of BWO;
- To solve the problem of local optima caused by the imbalance between the exploration phase and the exploitation phase in the original BWO algorithm, we added Cauchy mutation & Tent chaotic mapping (CMT) strategy in the exploitation phase of BWO algorithm to enhance the algorithm’s ability to jump out of local optima; we added the Firefly Algorithm (FA) strategy to the exploration phase to enhance the randomness and diversity of exploration, enhancing the exploration ability of the algorithm;
- We proposed an automated machine learning framework FAMBWO-MA-BiLSTM for ionospheric TEC prediction and optimization. In our framework, we first proposed a deep learning model for TEC prediction, named Multi-head Attentional Bidirectional Long Short-Term Memory (MA-BiLSTM). Then we use FAMBWO to optimize four hyperparameters of MA-BiLSTM, including learning rate, dropout ratio, batch size, and the number of neurons in MA-BiLSTM’s BiLSTM layer.

The paper is structured as follows. Section 2 introduces the literature review in TEC prediction and hyperparameter optimization. Section 3 introduces the original BWO algorithm. Section 4 introduces 3 strategies used to improve BWO and the improved FAMBWO algorithm. Section 5 presents experimental results and analysis. Section 6 introduces the FAMBWO-MA-BiLSTM framework for ionospheric TEC prediction and optimization. Section 7 summarizes the entire paper.

## 2. Literature Reviews

At present, deep learning models are the most popular tools in TEC prediction. The hyperparameter optimization methods for TEC prediction models mainly include manual setting and grid search.

The manual setting method is for researchers to manually set hyperparameters based on their own experience. For example, Maria Kaselimi et al. proposed an LSTM model for TEC prediction. Their model consisted of two bidirectional LSTM layers. The number of neurons in each LSTM layer was manually set to 60 and 72; The learning rate and batch size were manually set to 0.0001 and 28 [30]. Xu Lin et al. used a spatiotemporal network ST-LSTM to predict global ionospheric TEC. The number of convolutional kernels and the size of convolutional kernels were set to 64 and 5; The initial learning rate was set to 0.001 [31]. Xia, G. et al. [32] proposed an ionospheric TEC map prediction model named CAiTST. The bath size and learning rate were manually set to 32 and 0.001, respectively; The number and size of convolutional kernels to 40 and 5. In [33], Xia, G. et al. proposed the ED-ConvLSTM model to predict global TEC maps, where hyperparameters such as convolutional kernel size was manually set to 5, batch size was 32, and learning rate was 0.001. Xin Gao et al. proposed a TEC map prediction model based on multi-channel ConvLSTM. In their work, the batch size was manually set to 15, the learning rate dynamically decayed, and the decay rate of the learning rate was also manually set [34]. Huang, Z. et al. [35] applied ANN to predict the vertical TEC of a single station in China. The hyperparameters such as learning rate and crossover probability were manually set to 0.1 and 0.4, respectively. Liu, L. et al. proposed the ConvLSTM model for storm-time high-latitude ionospheric TEC maps prediction. The learning rate and batch size in their research were manually set to 0.00003 and 14, respectively [36]. In [37], Liu, L. et al. proposed the ConvLSTM model to predict global ionospheric TEC, in which the dropout, learning rate, and batch size were manually set to 0.2, 0.00005, and 72, respectively. Manual setting of hyperparameters is easily influenced by personal subjective opinions as it is based on the experience and intuition of researchers. Especially, many hyperparameters are continuous, and the hyperparameters that researchers manually set are almost impossible to be the optimal ones. That is to say, the model with manual hyperparameters cannot achieve its optimal performance.

The grid search method is an automatic hyperparameter optimization algorithm. It first discretizes each hyperparameter to form a discretized hyperparameter space. Then it exhaustively searches through all possible hyperparameter combinations in the discretized hyperparameter space to find the optimal ones. The grid search method solves the problem of excessive reliance on researchers’ experience and is applied to optimize TEC prediction models. For example, Tang J. et al. [38] proposed the CNN-LSTM-Attention model to predict ionospheric TEC, and the hyperparameters such as batch size, epochs, filters and kernel size in their model were determined by grid search method. Lei, D. et al. [39] proposed Attentional-BiGRU to predict ionospheric TEC. In their work, the range of batch size was set to {16, 32, 64, 128}, and the range of learning rate was discretized to {0.1, 0.05, 0.01, 0.005, 0.001}. Then, grid search method was used to search for the optimal hyperparameter combination within the given range. Tang J. et al. [40] proposed the BiConvGRU model to predict TEC in China, where the number of layers for BiConvGRU, the convolutional kernel size, and the learning rate were determined by the grid search method. Although the grid search method can automatically search for hyperparameters, it also has shortcomings. On the one hand, the grid search method is an exhaustive method, and when there are a large number of hyperparameters to be optimized, the computational cost is very expensive [41]. On the other hand, when using the grid search method, continuous hyperparameters will be discretized to form a discrete search space. However, not all values of continuous hyperparameters are included in the discretized search space. Therefore, the grid search method can only obtain suboptimal results, and it is almost impossible to find the optimal hyperparameters [42].

In other application fields of deep learning models, random search algorithms, Bayesian optimization methods, and swarm intelligence optimization techniques have also been applied to the hyperparameter optimization of deep learning models. The random search algorithm [43] randomly generates solutions and evaluates them to find the best one. Its time complexity is lower than that of grid search, but due to random selection, it may lead to unstable results, missing important hyperparameters. Furthermore, the random search method cannot learn from past iterations. Bayesian optimization [44] method uses probability models to learn from previous attempts and guides the search towards the optimal combination of hyperparameters in the search space. Compared with memoryless grid search and random search methods, Bayesian optimization can find better parameters in fewer iterations, but the proxy function selected in the probabilistic proxy model needs to rely on experience. Recently, swarm intelligence has been widely used for hyperparameter optimization, replacing outdated manual setting method and grid search method. For example, Maroufpoor, S. et al. [43] applied the Grey Wolf Optimization (GWO) algorithm to optimize the hyperparameters of artificial neural network (ANN) for reference evapotranspiration estimation. Compared with manual optimization algorithms, GWO improves ANN’s prediction accuracy by 2.75%. Ofori-Ntow Jnr et al. [44] proposed a short-term load forecasting method based on ANN and used Particle Swarm Optimization (PSO) to optimize its hyperparameters. The results showed that after using PSO optimization, the performance of ANN was improved by 7.3666%. P. Singh et al. [45] proposed a multi-layer particle swarm optimization (MPSO) algorithm to optimize hyperparameters of convolutional neural networks (CNN). Their research showed that the model optimized by MPSO had an accuracy improvement of 31.07% and 8.65% on the CIFAR-10 data set and CIFAR-100 data set, respectively, compared to the manually optimized model. Ling Chen, H. et al. proposed an improved PSO optimization algorithm (TVPSO) to optimize SVM. Their work showed that compared to manually optimized methods, TVPSO has improved SVM by 1.1% and 2.4% on the Wisconsin data set and German data set [46]. Shah, H. et al. used ant colony optimization (ACO) algorithm to optimize the BP neural network and reduced MSE of BP by 5.42% compared to the manual method [47]. Swarm intelligence has made some progress in many application fields such as machine learning and deep learning, but there are no relevant reports on its application in TEC prediction. At present, the hyperparameter optimization in TEC prediction still uses the most primitive and clumsy grid search method and manual tuning method, greatly limiting the TEC prediction performance.

## 3. Overview of Original BWO

The Beluga Whale Optimization (BWO) algorithm [29] is a swarm intelligence algorithm for solving optimization problems. It imitated the behaviors of beluga whales such as swimming, preying and whale fall. BWO includes exploration and exploitation phases. Beluga whales are used as the search agent, and each beluga whale is a candidate solution for a hyperparameter combination that is updated during the optimization. The beluga whale with the best fitness value corresponds to the optimal hyperparameter combination. The implementation process of the original BWO algorithm is as follows.

### 3.1. Initialization

Suppose the entire population has n individual beluga whales (i.e., n possible candidate solutions), the problem to be optimized is d-dimensional (i.e., the number of hyperparameters to be optimized is d). Firstly, the matrix of search agent positions is initialized randomly by Equation (1).

(1) $$X=\left[\begin{array}{c} x_{1}\\ x_{2}\\ \begin{array}{c} x_{3}\\ .\\ \begin{array}{c} .\\ x_{n} \end{array} \end{array} \end{array}\right]=\left[\begin{array}{cccc} x_{1,1} & x_{1,2} & \ldots & x_{1,d}\\ x_{2,1} & x_{2,2} & \ldots & x_{2,d}\\ x_{3,1} & x_{3,2} & \ldots & x_{3,d}\\ . & . & . & .\\ . & . & . & .\\ x_{n,1} & x_{n,2} & \ldots & x_{n,d} \end{array}\right]$$

where $x_{i}$ = [$x_{i,1}$, $x_{i,2}$, …, $x_{i,d}$] (i = 1,2, …, n) represents the position of the i-th individual beluga whale, which is the i-th possible optimal parameter combination. $x_{i,j}$ represents the *j*-th hyperparameter to be optimized for the i-th beluga whale.

During the optimization process, a fitness function F($x_{i}$) (corresponding to the objective function of the model to be optimized) is used to estimate the fitness value of beluga whale i, and the fitness values of all beluga whales are collected and stored in the fitness matrix $F_{X}$. The fitness matrix is as Equation (2).

(2) $$F_{X}=\left[\begin{array}{c} F\left(x_{1,1},x_{1,2},\ldots ,x_{1,d}\right)\\ F\left(x_{2,1},x_{2,2},\ldots ,x_{2,d}\right)\\ \vdots \\ F\left(x_{n,1},x_{n,2},\ldots ,x_{n,d}\right) \end{array}\right]$$

Sort all the fitness values, and the position of the beluga whale with the minimum fitness is the optimal hyperparameter.

To balance exploration and exploitation, the BWO algorithm adopts a balance factor $B_{f}$, which is calculated as Equation (3).

(3) $$B_{f}=B_{0}\left(1-T/2T_{max}\right)$$

where T is the current iteration, and $T_{max}$ is the maximum number of iterations, $B_{0}$ is a random parameter between 0 and 1. When $B_{f}$ > 0.5, the optimization algorithm enters the exploration phase, and when $B_{f}$ < 0.5, it enters the exploitation phase.

### 3.2. Exploration

The Beluga Whale Optimization algorithm provides two position update formulas during the exploration phase, as shown in Equation (4).

(4) $$\left\{\begin{array}{c} x_{i,j}^{T+1}=x_{i,p_{j}}^{T}+\left(x_{r,p_{1}}^{T}-x_{i,p_{j}}^{T}\right)\left(1+r_{1}\right)\sin\;\left(2\pi r_{2}\right),\;\;\;\;j=even\\ x_{i,j}^{T+1}=x_{i,p_{j}}^{T}+\left(x_{r,p_{1}}^{T}-x_{i,p_{j}}^{T}\right)\left(1+r_{1}\right)\cos\;\left(2\pi r_{2}\right),\;\;\;\;j=odd \end{array}\right.$$

where T is the current iteration, $x_{i,j}^{T+1}$ is the new position of the i-th beluga whale in the j-th dimension during the T+ 1 iteration. $p_{j}\left(j=1,2,3,...,d\right)$ is a random integer between 1 and d, $x_{i,p_{j}}^{T}$ indicates that in the T-th iteration, the position of the i-th beluga whale in dimension $p_{j}$. r is a random integer between 1 and n, $r_{1}$ and $r_{2}$ are both random numbers between 0 and 1.

### 3.3. Exploitation

In the exploitation phase, the Levy flight strategy is added to the BWO algorithm to accelerate its convergence speed and enhance its local search ability. The positions for beluga whales during the exploitation phase are updated as Equation (5).

(5) $$x_{i}^{T+1}=r_{3}x_{\mathrm{best}\;}^{T}-r_{4}x_{i}^{T}+C_{1}\cdot L_{F}\cdot \left(x_{r}^{T}-x_{i}^{T}\right)$$

where $x_{i}^{T+1}$ and $x_{i}^{T}$ represent the position of the beluga whale during iteration T + 1 and T, respectively. $x_{\mathrm{best}\;}^{T}$ is the optimal position during the T-th iteration. $x_{i}^{T}$ represents the position of a random beluga whale during the T-th iteration, $C_{1}=2r_{4}\left(1-T/T_{max}\right)$, is weight of Levy flight, $r_{3}$ and $r_{4}$ are random numbers between (0,1). $L_{F}$ is Levy flight function, which is calculated by Equations (6) and (7).

(6) $$L_{F}=0.05\times \frac{u\times \sigma }{|v{|}^{1/\beta }}$$

(7) $$\sigma ={\left(\frac{\Gamma \left(1+\beta \right)\times \sin\;\left(\pi \beta /2\right)}{\Gamma \left((1+\beta )/2\right)\times \beta \times 2^{(\beta -1)/2}}\right)}^{1/\beta }$$

where u and v represent random numbers with a normal distribution, and β is a constant. In the original BWO, β was 1.5.

### 3.4. Whale Fall

The whale fall phase simulates the process of a dead beluga whale falling into the seabed. Introducing this phase can enhance the algorithm’s ability to jump out of local optima. During the whale fall phase, the formula for updating the position of the beluga whale is defined as follows:

(8) $$x_{i}^{T+1}=r_{5}x_{i}^{T}-r_{6}x_{r}^{T}+r_{7}x_{step\;}$$

(9) $$x_{step}=\left(u_{b}-l_{b}\right)\exp\;\left(-C_{2}T/T_{max}\right)$$

where $r_{5},r_{6},r_{7}$ are random numbers between (0,1), $u_{b}$ and $l_{b}$ represent the upper and lower boundaries of the optimized parameters, $x_{step}$ is the step of whale fall. $C_{2}=2W_{f}\times n$, which is a parameter related to the step of whale fall step. $W_{f}$ is the probability of a whale falling, calculated using Equation (10).

(10) $$W_{f}=0.1-0.05T/T_{max}$$

The pseudo code of the original BWO optimization algorithm is shown in Algorithm 1.
**Algorithm 1.** Pseudocode of the original BWO**Input:**Parameters of BWO, such as $T_{max}$, the number of beluga whales n, the number of hyperparameters to be optimized d, and the upper and lower boundary of the parameters to be optimized, represented as $u_{b}$ and $l_{b}.$
**Output:**The best solution *P* *.1:Randomly initialize the population, calculate fitness values, and then find the current best solution.2:**while** $T\leq T_{max}$ **do**3:  Calculate the current probability of whale fall $W_{f}$ through Equation (10), and the current balance factor $B_{f}$ through Equation (3).4: **for** each candidate solution **do**5:  **if** $B_{f}\left(i\right)$ > 0.5 **then**6:   // In the exploration phase of BWO7:    Generate $p_{j}$(j=1,2,…,d) randomly8:     Select a beluga whale $x_{r}$ randomly9:     Update the position of the *i*-th beluga whale according to Equation (4)10:    **else if** $B_{f}\left(i\right)$ ≤ 0.511:     //In the Exploitation of BWO12:     Calculate the weight of Levy flight $C_{1}$,then calculate the Levy      flight function by Equations (6) and (7)13:     Update the position of the *i*-th beluga whale according to Equation (5)14:     **end if**
15:     Check the boundaries of new positions and evaluate the fitness16:  **end for**
17:  **for** each candidate solution ($x_{i}$) **do**18:     // In whale fall of BWO19:    **if**
$B_{f}\left(i\right)$ $\leq W_{f}$20:     Update the step factor $C_{2}$
21:     Calculate the whale fall step $x_{step}$ by Equation (9)22:     Update the position of the *i*-th beluga whale according to     Equation (8)23:     Calculate fitness based on the updated position of the beluga whale.24:    
**end if**
25:  
**end for**
26:  Find the current best solution *P* *27:  *T* = *T* + 128:**end while**29:Output the best solution

## 4. Our Improved BWO

Although the BWO algorithm has achieved some results in machine learning and deep learning hyperparameter optimization, the original BWO still has shortcomings such as insufficient initial population diversity and imbalanced development and exploration stages, making it easy for the Beluga algorithm to fall into local optima during hyperparameter optimization [48]. In order to solve the above problems, this paper has made three improvements to the BWO algorithm, including:

- Add cat chaotic mapping strategy (CCM) in the population initialization phase to increase population diversity;
- Add firefly algorithm (FA) strategy in the exploration phase to help it find the global optimal solution more easily;
- Add a CMT strategy (Cauchy Mutation and Tent chaotic) in the exploitation phase to enhance the algorithm’s ability to optimize nonlinear functions and jump out of local optima. We name the improved model FAMBWO.

Next, we will elaborate on the principles of the strategies used in this paper.

### 4.1. Cat Chaotic Mapping Strategy (CCM)

Cat chaotic mapping has good chaotic characteristics [49]. In order to solve the problem of insufficient diversity in the original BWO, this paper applies cat chaotic mapping strategy to replace the random initialization population method during population initialization phase. The steps to apply cat mapping chaos strategy to initial population are as follows:

- Firstly, randomly generate two d-dimensional vectors, $x_{1}$ = [$x_{1,1}$, $x_{1,2}$, … $x_{1,d}$], $y_{1}$ = [$y_{1,1}$, $y_{1,2}$, …, $y_{1,d}$], with each element’s between 0 and 1;

- Calculate n chaotic variables through cat mapping in Equation (11);

(11) $$\left[\begin{array}{c} x_{i+1}\\ y_{i+1} \end{array}\right]=\left[\begin{array}{c} 1\;\;\;\;1\\ 1\;\;\;\;2 \end{array}\right]\cdot \left[\begin{array}{c} x_{i}\\ y_{i} \end{array}\right]mod\;1,\;\;i=1,2,\ldots ,n$$

where $x_{i}$ *mod* 1 = $x_{i}$ − [$x_{i}$], $x_{i}$ = [$x_{i,1}$, $x_{i,2}$, …, $x_{i,d}$] (*i* = 1,2, …, *n*).

- Map chaotic variables to the range of parameters to be optimized using Use Equation (12).

(12) $${\;\;\;\;x}_{i}=l_{b}+\left(u_{b}-l_{b}\right)x_{i},i=1,2,\ldots ,n$$

where $u_{b}$ and $l_{b}$ represents the upper and lower boundaries of the parameters to be optimized.

### 4.2. Firefly Algorithm Strategy (FA)

The BWO optimization algorithm adopts two fixed position update formulas during the exploration phase, which limits its exploration performance. To solve this problem, we added an additional firefly algorithm strategy after the location update of the beluga whale, adding disturbance to the location update, increasing the diversity of location updates, and improving the exploration ability of the algorithm. In [50], it was pointed out that FA can enhance the ability of optimization algorithms to find global optima by simulating the behavior of fireflies emitting light to attract peers for information transmission. In FA, first calculate the spatial distance between two fireflies, then calculate the attraction between these two fireflies based on their distance, and finally update the position of the fireflies according to the attraction. The formula for calculating the spatial distance $r_{ir}$ between two fireflies $x_{i}$ and $x_{r}$ is shown in Equation (13).

(13) $$r_{ir}=∥x_{i}-x_{r}∥=\sqrt{\sum_{k=1}^{k=d}\;{\left(x_{i,k}-x_{r,k}\right)}^{2}\;}$$

The calculation method for the attraction $\beta (r_{ir})$ between $x_{i}$ and $x_{r}(i,r$ = 1, 2, …, n) is as Equation (14).

(14) $$\beta (r_{ir})=\beta_{0}e^{-\gamma r_{ir}^{2}}$$

where $\beta_{0}$ is the attraction of two fireflies at a distance of 0.

When firefly $x_{i}$ is attracted to firefly $x_{r}$, the position for $x_{i}$ is updated according to Equation (15).

(15) $$x_{i}=x_{i}+\beta (r_{ir})\left(x_{j}-x_{r}\right)+\alpha (r_{8}-0.5)$$

where $r_{8}$ is a random number within [0,1], α is a step factor between [0,1].

### 4.3. CMT Strategy

In order to improve the exploitation ability of BWO, we add a CMT strategy (Cauchy Mutation and Tent chaotic) in the exploitation phase. The CMT strategy is a combination of Cauchy mutation strategy and Tent chaotic mapping strategy.

#### 4.3.1. Cauchy Mutation Strategy

The Cauchy distribution has the characteristic of a long tail. Adding variables that follow the Cauchy distribution in position updates is called Cauchy variation, which can generate significant changes in the search space, helping the algorithm jump out of local minima and search globally. The Inverse Cumulative Distribution Function (ICDF) is used to generate random variables that follow the Cauchy distribution, and its definition is as Equation (16).

(16) $$F^{-1}\left(p;x_{0},\gamma \right)=x_{0}+\gamma \cdot tan\;\left(\pi \cdot \left(p-\frac{1}{2}\right)\right)$$

Inspired by ICDF, we propose a position updating formula for beluga whales based on a Cauchy mutation, and its calculation method is Equation (17).

(17) $$x_{i}=x_{i}+x_{i}\cdot \vec{\Lambda }\cdot tan\;\left(\pi \cdot \left(r_{9}-\frac{1}{2}\right)\right)$$

where $\vec{\Lambda }$ is a spiral factor to adjust the magnitude of the mutation operation and $r_{9}$ is a random number between [0, 1].

#### 4.3.2. Tent Chaotic Mapping Strategy

The Tent chaotic mapping has traversal uniformity and faster search speed. Using Tent chaotic mapping for optimization can improve the algorithm’s optimization ability for nonlinear problems and improve its accuracy [51]. The calculation formula for Tent chaotic mapping is shown in Equation (18).

(18) $$x_{n+1}=\left\{\begin{array}{cc} 2x_{n}, & 0\leq x_{n}\leq 0.5\\ 2\left(1-x_{n}\right), & 0.5\leq x_{n}\leq 1 \end{array}\right.$$

After adding the Tent chaotic mapping, the update formula for the position of the beluga whale is as Equation (19).

(19) $${\;x\;}_{i}=x_{i}+x_{i}\cdot x_{n+1}$$

where $x_{n}$ is a random number between [0, 1].

#### 4.3.3. CMT

The previous section described Cauchy mutation and tent chaotic mapping, and this section combines the two to propose a CMT strategy.

Let $F\left(x_{i}\right)$ be the fitness corresponding to the position of the *i*-th beluga whale, $F_{mean}$ be the average fitness of the population. In the CMT strategy, when $F\left(x_{i}\right){\leq F}_{mean}$, update the positions of beluga whales by Cauchy mutation strategy in Equation (17) so as to enhance the algorithm’s ability to jump out of local optima; otherwise, update the positions by Tent chaotic mapping strategy Equation (19) so as to increase the algorithm’s ability to optimize nonlinear functions.

### 4.4. The Details of Our Proposed FAMBWO

The three strategies used in this paper were presented earlier. In this section, we will combine these three strategies with BWO and describe in detail our proposed FAMBWO algorithm.

**Figure 1:**
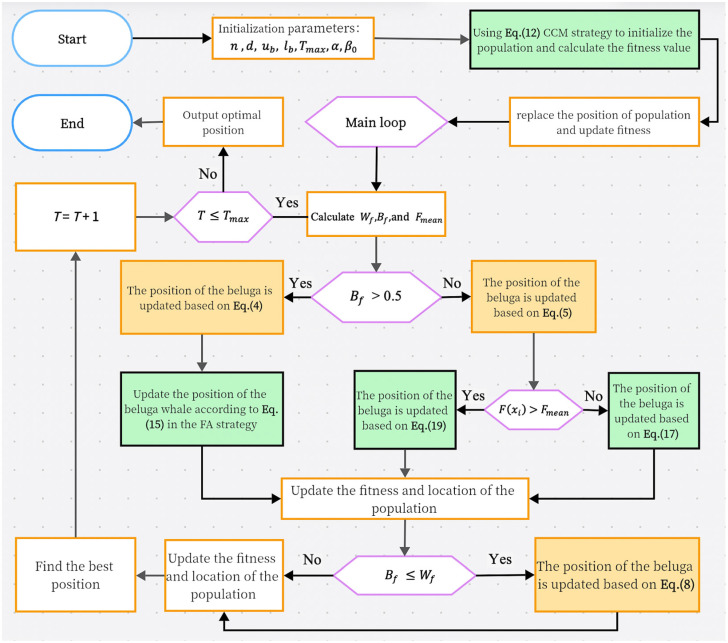
Flowchart of FAMBWO.

Our FAMBWO consists of three phases: initialization phase, exploration phase, and exploitation phase. The pseudo code of the FAMBWO algorithm is shown in Algorithm 2, and the flowchart is shown in Figure 1, where the green parts are our improvements.

Initialization phase: In our FAMBWO algorithm, the CCM strategy introduced in Section 4.1 is used to initialize the population, increasing its diversity and improving search efficiency.

Exploration phase: update the population with the FA strategy introduced in Section 4.2, improving the algorithm’s exploration ability and helping the algorithm find the global optimal solution more easily.

Exploitation phase: Update the population with the CMT strategy introduced in Section 4.3, improving the algorithm’s ability to optimize nonlinear functions and jump out of local optima.

We balanced the exploitation and exploration capabilities of the algorithm by combining FA and CMT strategies.

The pseudocode for FAMBWO is as Algorithm 2.
**Algorithm 2.** Pseudocode for FAMBWO**Input:**The initial parameters of FAMBWO, including $T_{max}$, n,
$d,u_{b}$ and $l_{b}$.**Output:**The best solution *P* *.1:Initialize the population through Equations (11) and (12).2:Calculate the fitness value and then find the location of the current best solution.3:**while** $T\leq T_{max}$ **do**4:Calculate the current probability of whale fall $W_{f}$ by Equation (10) and the current balance factor $B_{f}$ by Equation (3).5:Initialize parameters α, $\beta_{0}\;\mathrm{and}\;r_{8}$ in the firefly algorithm6:  **for** each begula $x_{i}$
**do**7:   **if** $B_{f}\left(i\right)$ > 0.5 **then**8:      // In exploration phase of FAMBWO9:      Randomly generate $p_{j}$ (*j* = 1, 2, …, d)10:      Randomly choose a beluga whale
$x_{r}$
11:      Update the position of *i*-th beluga whale by Equation (4)12:      Calculate the spatial distance $r_{ir}$ between $x_{i}$ and $x_{r}$ by Equation (13) and the attraction $\beta (r_{ir})$ between $x_{i}$ and $x_{r}$ by Equation (14)13:      Update the position of *i*-th beluga whale by Equation (15)14:   **else if** $B_{f}\left(i\right)$
≤ 0.515:      //In exploitation of FAMBWO16:      Calculate the random jump intensity factor $C_{1}$, and calculate   Levy flight function by Equation (6)17:      Update the position of *i*-th beluga whale by Equation (5)18:      
**if** 
$F\left(x_{i}\right){\leq F}_{mean}\;$
**then**
19:        Randomly generate *r*20:         Update the position of *i*-th beluga whale by Equation (17)     // cauchy mutation21:      **else if** $F\left(x_{i}\right)\;$>${\;F}_{mean}$22:         Calculate $x_{n+1}$ by Equation (18)23:         Update the position of *i*-th beluga whale by Equation (19)24:      
**end if**
25:   
**end if**
26:   Check the updated position of the beluga whale and calculate its fitness.27:  **end for**
28:  **for** each candidate solution **do**29:   **if** $B_{f}\left(i\right)$ > 0.5 **then**30:      // In whale fall of FAMBWO31:        Update the step factor
$C_{2}$
32:        Calculate the whale fall step $x_{step}$ by Equation (9)33:        Update the position of *i*-th beluga whale by Equation (8)34:        Check the updated position of the beluga whale and calculate        its fitness.35:      
**end if**
36:  end for37:  Find the best candidate solution for the current iteration *P* *38:  *T* = *T* + 139:**end while**40:Output the best solution

### 4.5. Computational Complexity

The time complexity of FAMBWO mainly includes population initialization, fitness evaluation, and population update. The main parameters that affect time complexity are the maximum number of iterations $T_{max}$, dimension of the problem *d*, and population size *n*. The time complexity of population initialization, computational fitness, and population update are O(*n* × *d*), O($T_{max}$ × *n*) and O($T_{max}$ × *d* × *n*), respectively. So, O(FAMBWO) = O(population initialization) + O(fitness evaluation) + O(population update) ≈ O(*n* × *d*) + O($T_{max}$ × *n*) + O($T_{max}$ × *d* × *n*) = O($T_{max}$ × *d* × *n*).

## 5. Experimental Results and Discussion

The performance of our proposed FAMBWO is evaluated on 30 well-known benchmark problems, and the results are compared with other 11 metaheuristic algorithms. In this section, we first introduced the benchmark problems and experimental setup, followed by discussing the influence of the 3 strategies, and then compared the exploitation ability, exploration ability, and local optimal avoidance ability of our algorithm with the other 11 mainstream metaheuristic optimization algorithms. In addition, scalability analysis was conducted on 12 algorithms to compare their ability to handle high-dimensional optimization problems.

### 5.1. Benchmark Problems and Experimental Setup

To evaluate the performance of the proposed FAMBWO, 30 different benchmark problems were chosen for comparative experiments, including 9 unimodal functions (F1–F9, as shown in Table A1 of Appendix A) for testing the exploitation ability, 15 multimodal functions (F10–F24, as shown in Table 2) for testing the exploration ability, and 6 composition functions (F25–F30, as shown in Table 3) for evaluating the local optimum avoidance ability. In Table 1, Table 2 and Table 3, Range represents the bound of design variable, and $f_{min}$ is the optimal value.

The comparison algorithms are 11 mainstream metaheuristic optimization algorithms, including PSO [20], MFO [21], DE, SCA [22], SSA [23], WOA [24], SOA [25], GWO [26], DBO [27], WSO [28], and BWO [29]. The parameters of the comparison algorithms are shown in Table 4.

During the experiment, the population size of each algorithm was 50 and the maximum number of iterations was 200. To eliminate the influence of random factors, each algorithm was independently executed 30 times on each benchmark function. The Friedman test method was used to rank the fitness of all the algorithms on the benchmark functions to evaluate their performance [52].

All algorithms were written in Python 3.7 and tested on a computer equipped with an Intel (R) Xeon (R) CPU E5-2686 v4 12 core processor and an NVIDIA GeForce RTX 3060 Ti graphics card with 8 GB VRAM.

**Table 4:** Algorithmic parameters for metaheuristics [53,54,55,56].

Algorithm	Parameters	Values
# All algorithms	Population size, maximum iterative number, replication times	50, 200, 30
PSO	Cognitive and social constant Inertia weight linearly decreased at interval	$c_{1}$ = 2, $c_{2}$ = 2 [0.9,0.2]
MFO	Convergence constant spiral factor	a = [−2 −1], *b* = 1
DE	Scaling factor, crossover probability	0.5, 0.5
SCA	spiral factor	[0 2]
SSA	Leader position update probability	0.5
WOA	Probability of encircling mechanism, spiral factor	0.5, 1
SOA	Control parameter $f_{c}$	[2,0], 2
GWO	Convergence parameter a decreased at interval	[2 0]
DBO	Special Parameters	*K* = 0.1, *b* = 0.3, and *S* = 0.5
WSO	Convergence constant	0.8
BWO	Probability of whale fall decreased at interval $W_{f}$	[0.1 0.05]
FAMBWO	$\beta_{0}$, step factor a, Probability of whale fall decreased at interval $W_{f}$, spiral factor $\vec{\Lambda }$	$\beta_{0}$ = 2, a = 0.2, $W_{f}$ = [0.1 0.05], $\vec{\Lambda }$ = [0 2]

### 5.2. Influence of the Three Strategies

In this section, CCM strategy, FA strategy, and CMT strategy are combined with BWO in different ways to analyze their impact on improving BWO performance. The details of these various BWOs are shown in Table 5, where ‘1’ indicates that the strategy is added to BWO and ‘0’ means vice versa.

Table A1 in Appendix A shows the results of these various BWOs on 30 benchmark problems, with Aver being the average and Std being the standard deviation.

According to the Aver of each algorithm in Table A1, we perform a Friedman test and obtain the ranking of each algorithm as shown in Table 6, with Rank indicating the algorithm’s rank, Avg being the average rank, and ‘+/−/=’ indicating the number of benchmark problems where FAMBWO’s performance is superior, inferior, or equal to other algorithms, respectively.

From Table 7, the performance of the 8 BWOs from best to worst is FAMBWO > CMT_FA_BWO > CCM_CMT_BWO > CCM_FA_BWO > FA_BWO > CMT_BWO > BWO > CCM_BWO. The FAMBWO algorithm, which includes CCM, FA, and CMT strategies, ranks first, indicating that adding these three strategies simultaneously to BWO can significantly improve algorithm performance.

### 5.3. Comparison with State-of-the-Art SI Algorithms

To evaluate the performance of our proposed FAMBWO, we compared it with 11 other mainstream swarm intelligence optimization algorithms on 30 benchmark problems. The comparison algorithms include PSO [20], MFO [21], DE, SCA [22], SSA [23], WOA [24], SOA [25], GWO [26], DBO [27], WSO [28], and BWO [29]. The comparative experiments are divided into five parts: Firstly, we analyzed the convergence behavior of FAMBWO; secondly, the exploitation abilities of all algorithms were compared on the unimodal function (F1–F9); thirdly, the exploration abilities of various algorithms were tested on multimodal functions (F10–F24); fourthly, the ability of local optimum avoidance was evaluated on composition functions (F25–F30); finally, scalability analysis was conducted on composition functions (F25–F30) of 100 dimensions to compare their ability to handle high-dimensional optimization problems. Below is a detailed discussion of these comparative experiments.

#### 5.3.1. Convergence Behavior Analysis

**Figure 2:**
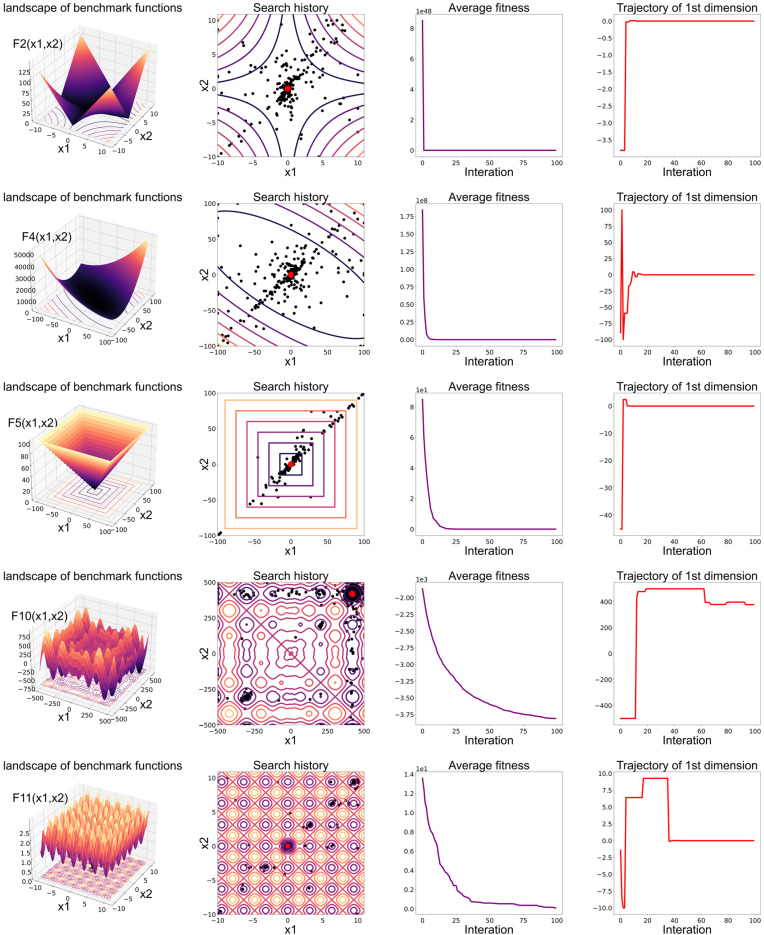

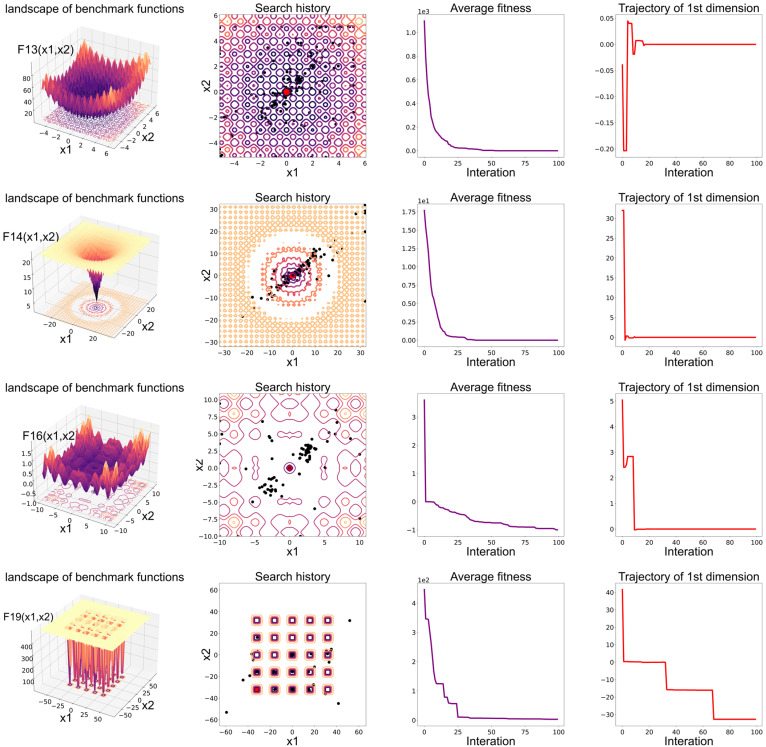
The convergence behavior of FAMBWO.

To validate whether FAMBWO converges, we tested its convergence behaviors on 10 benchmark functions, including 4 unimodal functions (F1, F2, F4, F5) and 6 multimodal functions (F10, F11, F13, F14, F16, F19). Results of convergence behaviors are presented in Figure 2, including: (1) landscape of benchmark functions; (2) the search history of search agents; (3) the average fitness of search agents; and (4) the trajectory of the first dimension.

The benchmark functions in Figure 2 were used as search spaces for FAMBWO. The global minimum value on each benchmark function is the ultimate best solution of FAMBWO.

The search history in Figure 2 showed the distribution of search agents’ positions in the process of finding the global optimal solution, with the red dot denoting the globally optimal solution and the black ones indicating the search agents’ positions. From the search history, it is clearly seen that on unimodal functions (F1, F2, F4, F5), the search trajectory clustered near the global best solution. This indicates that FAMBWO can achieve fast convergence. On 4 multimodal functions (F11, F13, F14, F16), the search history of FAMBWO shows a nearly linear pattern, indicating FAMBWO can avoid local optima and ensure the global solution. On F10 and F19, the search trajectory is concentrated near the optimum solution and distributed throughout the search space, indicating that FAMBWO can effectively explore the search space.

The third column in Figure 2 shows the change in the average fitness of the search agent. It can be seen that average fitness rapidly decreases during the initial stage of the iteration, indicating that FAMBWO can converge quickly.

The fourth column in Figure 2 shows the trajectory of the first search agent in the first dimension. It represents the primary exploratory behavior of FAMBWO. The results show that it fluctuates sharply in the early stages of the iteration and gradually stabilizes in the later stages, ensuring that FAMBWO can eventually converge.

#### 5.3.2. Exploitation Ability Analysis

**Figure 3:**
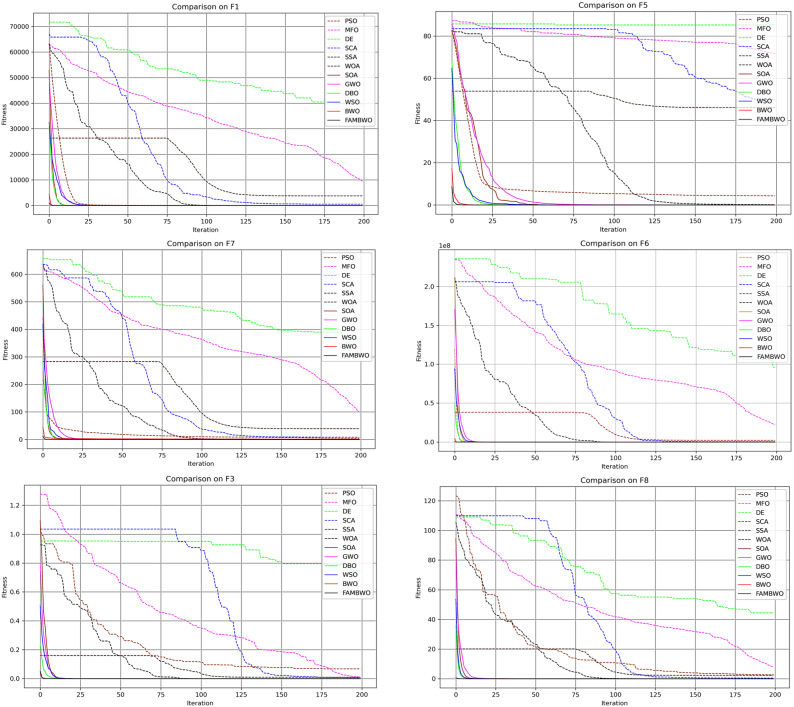
Convergence curves of different algorithms on the unimodal functions.

To verify the exploitation capability of our FAMBWO, we compared it with 11 metaheuristic algorithms on 9 unimodal functions (F1–F9). We select 6 unimodal test functions to present the convergence curves of 12 optimization algorithms, as shown in Figure 3. It can be seen that, compared with the other 11 algorithms, FAMBWO’s fitness changes rapidly and converges earliest in the initial stage of iteration, indicating that FAMBWO requires the least number of iterations to find the optimal solution and has the fastest convergence speed. It can also be seen that the position of the optimal solution of FAMBWO is the lowest, indicating that the FAMBWO algorithm has the highest accuracy.

The quantitative statistical results of the optimal fitness of 12 algorithms on the unimodal benchmark function are shown in Table A2 of Appendix A, with Aver and STD being the mean and standard deviation of fitness. FAMBWO ranks first in terms of Aver and STD on all unimodal functions except F7 and is significantly superior to other comparison algorithms. On function F7, FAMBWO ranks second after WSO.

According to the Aver in Table A2, we perform the Friedman test on 12 algorithms, and the results are shown in Table 7, where Avg represents the average ranking of the algorithm in the test, and Rank represents the final ranking. The smaller the Rank and the Avg, the better the performance of the algorithm. We can see from Table 9, FAMBWO ranks first among the 12 algorithms.

**Table 7:** Friedman test results on unimodal functions (F1–F9, dim = 30).

Fun	Rank	Avg
FAMBWO	1	1.1111
PSO	9	8.4444
MFO	11	10.5556
DE	12	11.7778
SCA	8	8.1111
SSA	10	9.7778
WOA	6	5.7778
SOA	3	4.3333
GWO	5	4.7778
DBO	7	6.4444
WSO	4	4.3333
BWO	2	2.5556

Table A3 in Appendix A presents the Wilcoxon signed rank test results of the FAMBWO algorithm compared to other algorithms. *p*-value < 0.05 indicates that the FAMBWO algorithm has significant statistical advantages compared to other comparative algorithms. From Table 10, it can be seen that on the 9 unimodal functions, the vast majority of *p*-values are less than 0.05. Therefore, it can be concluded that FAMBWO is significantly superior to the other 11 comparative algorithms, indicating that FAMBWO’s exploitation ability is significantly better than those of the comparative algorithms.

#### 5.3.3. Exploration Analysis

**Figure 4:**
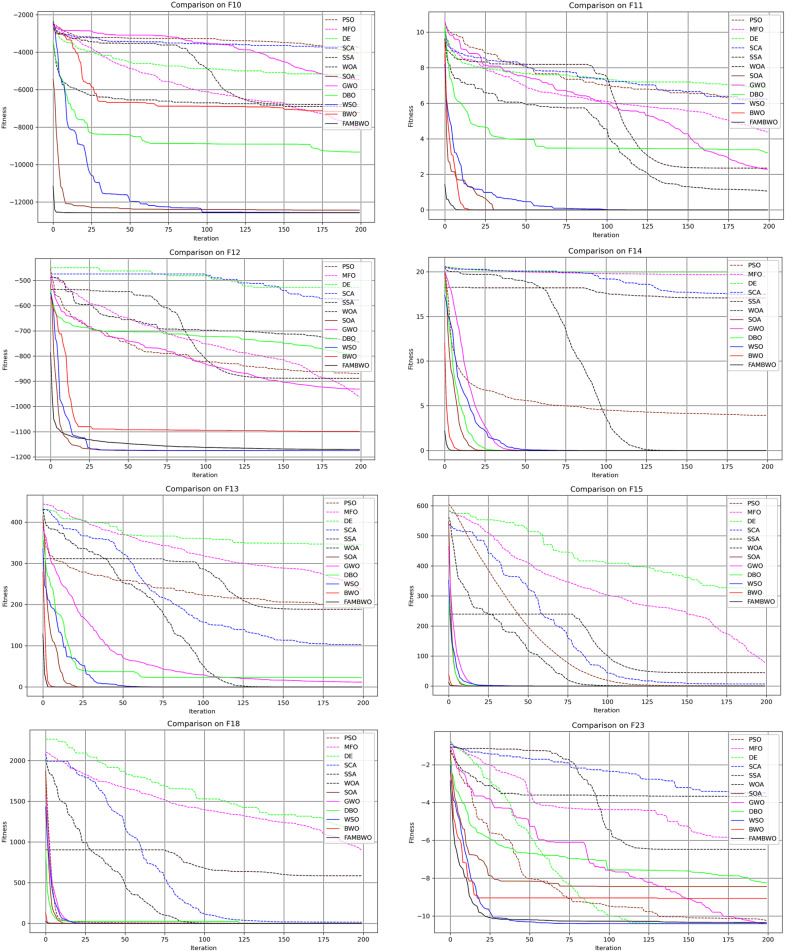

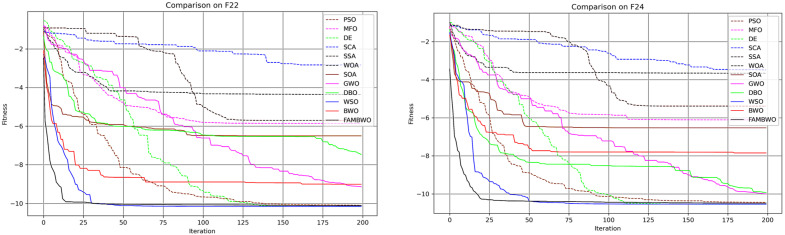
Convergence curves of different algorithms on the multimodal functions.

In the previous section, we evaluated the exploitation ability of algorithms on unimodal functions. In this section, we compare the optimization capabilities of algorithms on multimodal functions, which have many local optimal solutions that can be used to evaluate the exploration ability of the algorithm. We selected 15 multimodal functions (F10–F24) and conducted 30 independent experiments on each one. Table 11 shows the average optimal fitness values of 12 algorithms on these multimodal functions. We present convergence curves on 12 multimodal functions to demonstrate the exploitation ability, as shown in Figure 4. It can be seen that compared with the other 11 optimization algorithms, FAMBWO has the fastest convergence speed and the lowest position of the optimal solution. This indicates that FAMBWO has the fastest speed in exploring the optimal solution, and the solution found by FAMBWO is closest to the global optimal solution.

From Table A4 in Appendix A, we can see that FAMBWO ranks first on ten functions (F10–F11, F13–F19, and F21), second on one function (F20), third on three functions (F12, F22, and F23), and fourth on F24.

Table 8 shows the Friedman test results of Aver, indicating that FAMBWO ranks first among the 12 algorithms.

In addition, we conducted a Wilcoxon signed rank test based on the average fitness. Table A5 in Appendix A presents the Wilcoxon signed rank test results of the FAMBWO and other algorithms. Among them, the vast majority of *p*-values are less than 0.05. Therefore, it can be concluded that FAMBWO is significantly better than the other 11 comparison models, meaning that FAMBWO’s exploration ability is better than the comparison algorithms.

#### 5.3.4. Local Optimal Avoidance Ability

Composition functions combine the characteristics of multiple basic functions, making them more complex compared to unimodal or multimodal functions. They are typically used to test algorithms’ ability to jump out of local optima. We conducted comparative experiments on 6 composition functions (F25–F30) and tested the local optimal avoidance ability of 12 algorithms.

**Figure 5:**
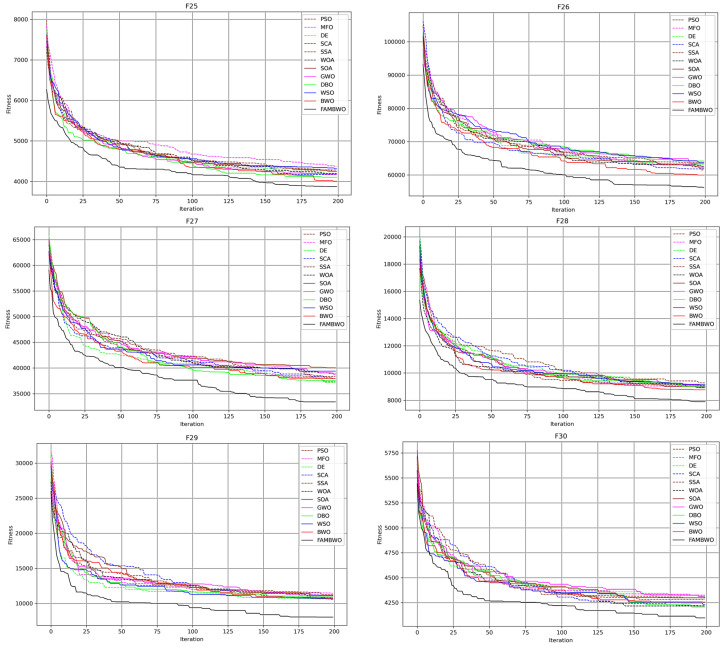
Convergence curves of different algorithms on composition functions with 30 dimensions.

Figure 5 shows the convergence curves of 12 optimization algorithms on 6 combination functions. Among them, we can see that the value of our FAMBWO’s optimal solution is smaller than those of other comparative models. This indicates that our algorithm outperforms the comparative algorithms in optimizing problems with multiple local optima. That is to say, our FAMBWO’s ability to avoid local minima exceeds that of the comparative algorithms.

The quantitative statistical results of the optimal fitness of 12 algorithms on the composition functions are shown in Table A6 of Appendix A. It can be seen that FAMBWO ranks first on all 6 composition functions.

We carried out the Friedman test according to the average fitness (Aver) in Table 9. The Friedman test results are shown in Table 9. It is easy to see that FAMBWO ranks first among the 12 algorithms.

We still conducted the Wilcoxon signed rank test according to the average fitness. Table A7 presents the Wilcoxon signed rank test results of the FAMBWO and other algorithms. From Table A7 in Appendix A, on the 15 multimodal functions, the vast majority of *p*-values are less than 0.05. Therefore, it can be concluded that FAMBWO’s ability to jump out of local optima is significantly better than the comparison algorithms.

#### 5.3.5. Scalability Analysis

**Figure 6:**
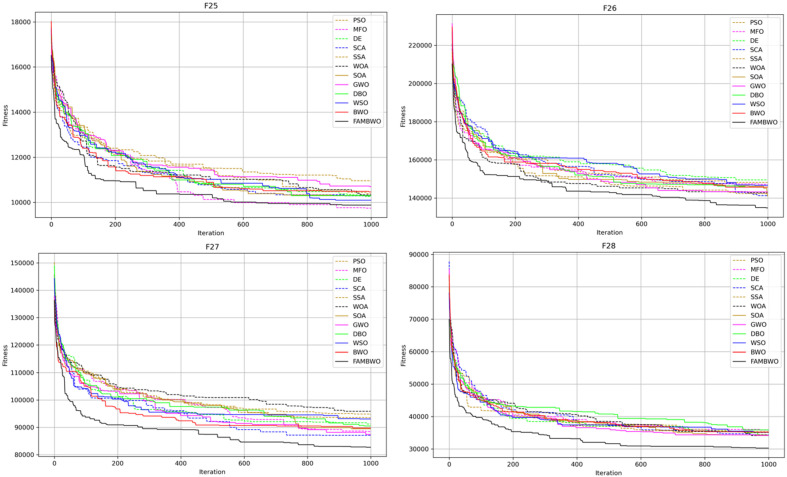

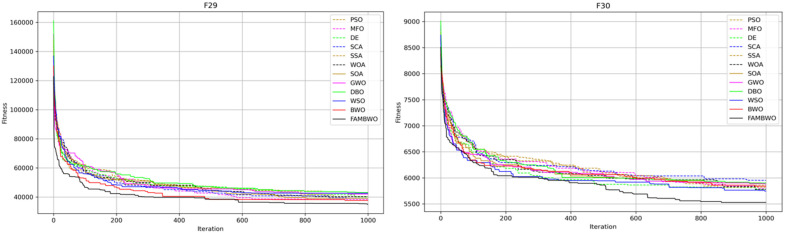
Convergence curves of different algorithms on composition functions with 100 dimensions.

The benchmark functions used in the previous experiments were all 30 dimensions. To test the ability of FAMBWO to solve high-dimensional optimization problems, we conducted scalability analysis on 6 composite functions (F25–F30) in 100 dimensions. During the experiment, the population size of each algorithm was 50, and the maximum number of iterations was 1000. Meanwhile, to eliminate the influence of random factors, each algorithm was independently executed 30 times on each benchmark function. The experimental results are shown in Figure 6:

From Figure 6, the convergence speed and optimal solution of FAMBWO are significantly better than those of the comparison models on F26–F30, slightly inferior to MFO on F25. This indicates that our FAMBWO outperforms the comparative algorithms in solving high-dimensional optimization problems.

Table A8 in Appendix A shows the statistical results (mean and standard deviation) of 12 algorithms on 100-dimensional combination functions F25–F30.

From Table A8, FAMBWO ranks first in the five combination functions (F26–F30) of 100 dimensions, only slightly inferior to the MFO on F25. The Friedman test results for the average fitness (Aver) on F25–F30 are shown in Table 10, from where we can see that FAMBWO ranks first in the 100-dimensional composition functions (F25–F30).

Table A9 in Appendix A shows the *p*-values of FAMBWO compared to 11 other algorithms on F25–F30. Among them, most *p*-values are less than 0.05, indicating that FAMBWO is significantly superior to the other 11 comparison algorithms in high-dimensional function optimization.

## 6. Optimizing the Ionospheric TEC Prediction Model Using FAMBWO

Previous experiments have been conducted on benchmark functions. In this section, we will apply our proposed FAMBWO to optimize practical application. We proposed a framework for ionospheric TEC prediction named FAMBWO-MA-BiLSTM. In this framework, we proposed a deep learning model based on Multi-Head Attention and BiLSTM for TEC prediction, which we named MA-BiLSTM. We then used FAMBWO to optimize the hyperparameters of MA-BiLSTM. We compared FAMBWO-MA-BiLSTM with GS-MA-BiLSTM (MA-BiLSTM optimized by grid search method), RS-MA-BiLSTM (MA-BiLSTM optimized by random search), BOA-MA-BiLSTM (MA-BiLSTM optimized by Bayesian optimization algorithm), and BWO-MA-BiLSTM (MA-BiLSTM optimized by BWO). The following describes the TEC data set and data preprocessing, MA-BiLSTM model, FAMBWO-MA-LSTM framework, and comparative experimental results.

### 6.1. Data Set and Data Preprocessing

**Figure 7:**
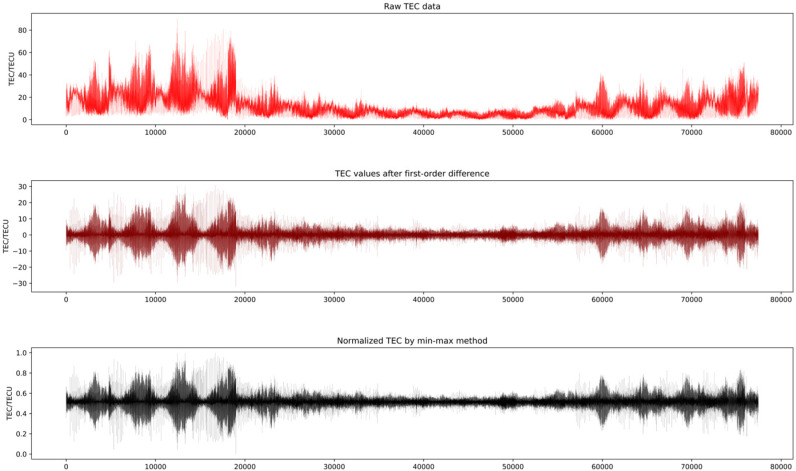
The raw and processed TEC: the upper shows the raw TEC, the middle shows the first-order difference, and the bottom shows the normalized TEC.

The TEC data used in this paper are provided by the Center for Orbit Determination in Europe (CODE), with a time resolution of 2 h. We selected TEC data from UT0:00 on 1 January 1999 to UT12:00 on 30 April 2015 at positions (25° N, 105° E) for the experiment. Our data include 77,467 TEC values. The raw TEC data are unstable and cannot be directly modeled. Therefore, we performed a first-order difference on the raw data to make them stationary. Then, we normalized them by the min-max method to eliminate the impact of data scale on prediction performance. The raw TEC and the processed TEC are shown in Figure 7.

**Figure 8:**
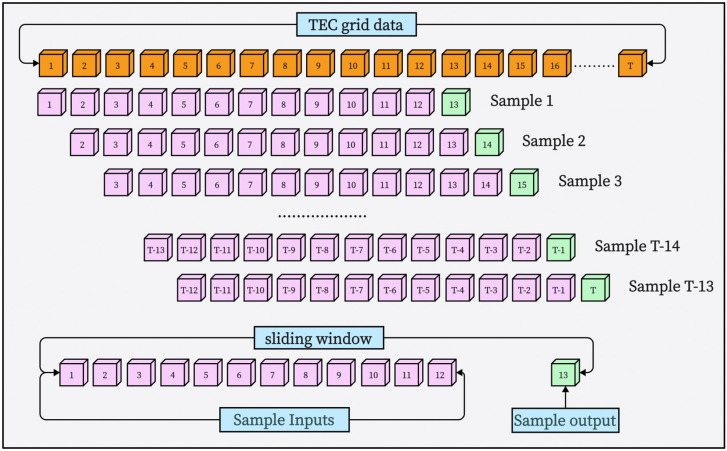
Schematic diagram of sample-making process.

In this paper, continuous 24-h TEC data are used as input to predict the next 2 h of TEC in the future. So, the input of a sample contains 12 TEC values, and the output contains 1 TEC value. We adopted a sliding window method to segment different samples, with each sliding for two hours. The sample production process is shown in Figure 8, with the purple data as input and the blue as output. In total, we obtained 77,454 samples, of which the samples from the first 14 years were used as training samples (1 January 1999 to 1 January 2013), and the ones from the rest were used as testing samples (1 January 2013 to 30 April 2015).

### 6.2. MA-BiLSTM

**Figure 9:**
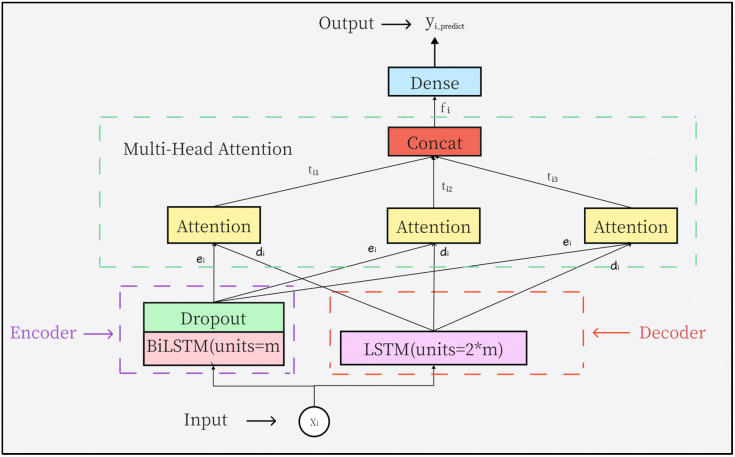
MA-BiLSTM model structure.

In this section, we proposed a TEC predicting model, named Multi-Head Attentional Bidirectional Long Short-Term Memory (MA-BiLSTM), which includes five modules: the input module, the encoder module, the decoder module, multi-head attention module and the output module. Its structure is shown in Figure 9.

**Input module**: used to receive samples. The input shape is (12,1), indicating 12 TEC values within 1 day.

**Encoder module:** This module contains a BiLSTM layer with *m* units (*m* is the hyperparameter to be optimized), and a Dropout layer with a ratio of *r* (*r* is the hyperparameter to be optimized). The encoder module is used to extract bidirectional temporal features from the input ${\;X}_{i}$, and the output of this module is $e_{i}$, representing the bidirectional temporal feature vector corresponding to ${\;X}_{i}$.

**Decoder module:** This module consists of 2*m LSTM units, with an output of $d_{i}$, used to assist in calculating the weights of temporal features.

**Multi-Head Attention module:** This module contains three independent attention heads, obtaining three weighted temporal features ($t_{i1},t_{i2},t_{i3}$), which are then connected to form the final weighted feature vector $f_{i}$. In this module, each attention head receives $e_{i}$ from the encoder module and $d_{i}\;$ from the decoder module and calculates their similarity score. The similarity score of the *j*-th attention head ${score}_{j}\;$($e_{i}$, $d_{i}$) by Equation (20).

(20) $${score}_{j}(e_{i},d_{i})=V_{j}^{T}tanh\;\left[{W_{j}e}_{i}+U_{j}d_{i}\right]\;\;\;\;\;(j=1,2,3)$$

where ${U_{j},V_{j},W}_{j}(j=1,2,3)$ are the parameters that can be learned in the training process. After obtaining the attention score, then normalize it with the softmax function to obtain the probability distribution of attention. The specific calculation formula is as Equation (21):

(21) $$a_{ij}=softmax\;\left(\mathrm{score}\;\left(h_{i},y_{i}\right)\right)=\frac{{score}_{j}(e_{i},d_{i})}{\sum e^{{score}_{j}(e_{i},d_{i})}}(j=1,2,3)$$

$a_{ij}$ represents the respective attention distribution value of the *j*-th attention head.

Then, $a_{ij}\;$ is multiplied by $e_{i}$ to obtain the weighted feature of the *j*-th attention head $t_{ij}$. The calculation of $t_{ij}$ is shown as Equation (22):

(22) $$t_{ij}=a_{ij}\times e_{i}(j=1,2,3)$$

Finally, connect the 3 weighted feature vectors from the 3 attention heads as the final weighted feature $f_{i}$. The calculation of $f_{i}$ is shown in Equation (23).

(23) $$f_{i}=[t_{i1},t_{i2},t_{i3}]$$

where [] represents the concatenation of vectors.

**Output layer:** This layer includes a fully connected layer (Dense). It is used to map the weighted temporal features $f_{i}$ into the predicted values and then output them.

### 6.3. FAMBWO-MA-BiLSTM Framework

When using MA-BiLSTM for TEC prediction, there are four important hyperparameters that affect its prediction performance, including the number of BiLSTM units, the proportion of dropouts, the batch size and the learning rate. We used FAMBWO to optimize these four hyperparameters. Firstly, the upper and lower boundaries of these four hyperparameters should be given to form the search space. The search space for these 4 hyperparameters is shown in Table 11. Secondly, initialize FAMBWO. Among them, the maximum number of evaluations *T_max_* is 200, the dimension *d* is 4, and the population size *n* is 30.

Then, the loss of MA-BiLSTM is used as the fitness function (in this paper, the loss function is MSE). The solver of MA-BiLSTM is set to AdaGrad. Finally, the FAMBWO algorithm is used to search for the optimal hyperparameters of MA-BiLSTM.

**Figure 10:**
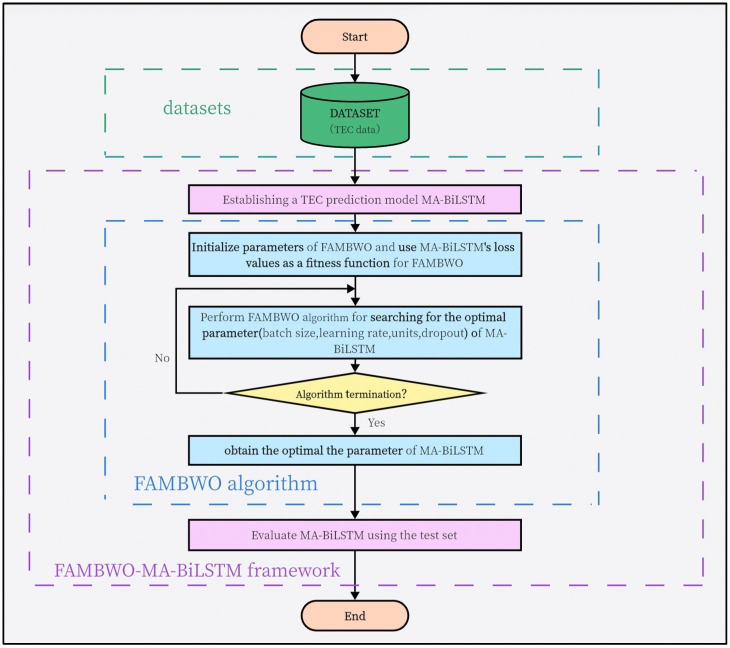
Flowchart of FAMBWO-MA-BiLSTM.

We name the entire framework for TEC modeling and optimization as FAMBWO-MA-BiLSTM, in which MA-BiLSTM is for TEC prediction and FAMBWO is for hyperparameters optimization. Figure 10 shows the flowchart of the entire FAMBWO-MA-BiLSTM framework.

### 6.4. Performance Metrics

The following metrics are used to quantitatively evaluate the predictive performance.

(24) $$MSE=\frac{1}{N}\sum_{i=1}^{N}{\left(Y_{i}-{\hat{Y}}_{i}\right)}^{2}$$

(25) $$RMSE=\sqrt{\frac{1}{N}\sum_{i=1}^{n}{\left(Y_{i}-{\hat{Y}}_{i}\right)}^{2}}$$

(26) $$MAE=\frac{1}{N}\sum_{t=1}^{N}\;∥Y_{i}-{\hat{Y}}_{i}∥$$

(27) $$R^{2}=\frac{\sum_{i=1}^{N}{\left(Y_{i}-{\hat{Y}}_{i}\right)}^{2}}{\sum_{i=1}^{N}{\left(Y_{i}-\bar{Y}\right)}^{2}}$$

(28) $$\bar{Y}=\frac{1}{N}\sum_{i=1}^{N}Y_{i}$$

where *N* is the number of samples in the test set; $Y_{i}$ is the true value of sample *i*; ${\hat{Y}}_{i}$ is the predicted value of the *i*-th sample; MSE is the mean-square error; RMSE is the root mean square error; MAE is the mean absolute error; $R^{2}$ is the correlation coefficient.

*MSE*, RMSE, and *MAE* reflect the errors between the true and predicted values, indicating how far the predicted values are from the true values. The smaller the error, the better the prediction performance of the model.$R^{2}$ describes the correlation between predicted values and true values. The larger the $R^{2}$, the higher the correlation between the predicted values and the true values.

### 6.5. Comparison Results on TEC Prediction

**Figure 11:**
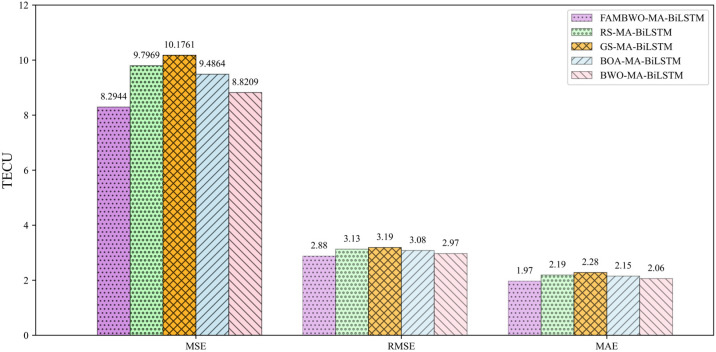
Comparison of prediction errors among 4 frameworks.

We compared the performance of the optimized MA-LSTM model using four optimization methods, namely grid search method, random search method, Bayesian optimization algorithm, and beluga optimization algorithm. The result is shown in Figure 11, where RS-MA-BiLSTM, GS-MA-BiLSTM, BOA-MA-BiLSTM, and BWO-MA-BiLSTM represent MA-BiLSTM model optimized by random search method, grid search method, Bayesian optimization algorithm, and beluga optimization algorithm, respectively. Compared to GS-MA-BiLSTM, our framework has reduced MSE by 18.50%, RMSE by 9.72%, and MAE by 13.60%. Compared to RS-MA-BiLSTM, our framework has reduced MSE by 15.38%, RMSE by 7.99%, and MAE by 10.05%. Compared to BOA-MA-BiLSTM, our framework has reduced MSE by 12.57%, RMSE by 6.49%, and MAE by 8.37%. Compared to BWO-MA-BiLSTM, our framework has reduced MSE by 5.98%, RMSE by 3.03%, and MAE by 4.37%. Table 12 presents the quantitative comparison results of the three frameworks. Obviously, FAMBWO-MA-BiLSTM is significantly better than RS-MA-BiLSTM, GS-MA-BiLSTM and BOA-MA-BiLSTM. Compared to BWO-MA-BiLSTM, our proposed framework also shows obvious improvement. These experimental results also show that simply optimizing hyperparameters can significantly improve the predictive performance of the model, indicating that hyperparameter optimization is even more important than model selection.

## 7. Conclusions

Deep learning is currently the state-of-the-art technology for TEC prediction, and hyperparameter optimization in deep learning models is a challenge, which greatly affects the performance of deep learning models. This article proposes a TEC prediction and optimization framework FAMBWO-MA-BiLSTM. We first analyzed the problems of the BWO algorithm, such as a lack of population diversity and an imbalance between the exploration and exploitation phases. We then proposed an improved algorithm FAMBWO by applying Cat chaotic mapping strategy during population initialization phase, adding Firefly Algorithm strategy in the location updating, and adding Cauchy mutation & Tent chaotic mapping strategy in the exploitation phase. We validated the effectiveness of adding these three strategies through ablation experiments. Then we compared our proposed FAMBWO with 11 other meta-heuristic algorithms on 30 benchmark functions, comparing their exploration, exploitation, and local optimal avoidance capabilities. The experimental results show that our proposed FAMBWO outperforms the comparative algorithms in terms of exploration ability, exploitation ability, local optimal avoidance ability, and the ability to solve high-dimensional optimization problems. Finally, we used the FAMBWO to solve the hyperparameter optimization problem of deep learning models in TEC prediction. We proposed an automated machine learning framework FAMBWO-MA-BiLSTM for TEC prediction and optimization. In this framework, MA-BiLSTM was used for TEC prediction and FAMBWO was used to optimize four hyperparameters of MA-BiLSTM. We compared our FAMBWO-MA-BiLSTM with GS-MA-BiLSTM, RS-MA-BiLSTM, BOA-MA-BiLSTM and BWO-MA-BiLSTM. The results indicate that the predictive performance of the FAMBWO-MA-BiLSTM framework is far superior to GS-MA-BiLSTM, RS-MA-BiLSTM, BOA-MA-BiLSTM and obviously outperforms BWO-MA-BiLSTM.

The study in this paper provides a new solution for deep learning hyperparameter optimization in TEC prediction and also provides reference for hyperparameter optimization in other deep learning application fields.

## Figures and Tables

**Table 1:** Details of benchmark problems for unimodal functions.

	Name	Function	Range	$f_{min}$
F1	Sphere	$\sum_{i=1}^{D}\;x_{i}^{2}$	[−100,100]	0
F2	Schwefel’s 2.22	$\sum_{i=1}^{D}\;\left|x_{i}\right|+\prod_{i=1}^{D}\;\left|x_{i}\right|$	[−10,10]	0
F3	Powell Sum	$\sum_{i=1}^{D}\;{\left|x_{i}\right|}^{i+1}$	[−1,1]	0
F4	Schwefel’s 1.2	$\sum_{i=1}^{D}\;{(\sum_{j=1}^{D}\;x_{j})}^{2}$	[−100,100]	0
F5	Schwefel’s	$\underset{i}{max}\;\left\{\left|x_{i}\right|,1\leq i\leq D\right\}$	[−100,100]	0
F6	Rosenbrock	$\sum_{i=1}^{D-1}\;[100{\left(x_{i+1}-x_{i}^{2}\right)}^{2}+{\left(x_{i}-1\right)}^{2}]$	[−30,30]	0
F7	Step	$\sum_{i=1}^{D}\;{\left(x_{i}+0.5\right)}^{2}$	[−100,100]	0
F8	Quartic	$\sum_{i=1}^{D}\;ix_{i}^{4}+\mathrm{random}\left[0,1\right)$	[−1.28,1.28]	0
F9	Zakharov	$\sum_{i=1}^{D}\;x_{i}^{2}+{(\sum_{i=1}^{D}\;0.5ix_{i})}^{2}+{(\sum_{i=1}^{D}\;0.5ix_{i})}^{4}$	[−5,10]	0

**Table 2:** Details of benchmark problems for multimodal functions.

	Name	Function	Range	$f_{min}$
F10	Schwefel	$-\sum_{i=1}^{n}\;\left(x_{i}\sin\sqrt{\left|x_{i}\right|}\right)$	[−500,500]	−418.98 × *d*
F11	Periodic	$1+\sum_{i=1}^{D}\;\sin^{2}\;\left(x_{i}\right)-\exp\;\left(\sum_{i=1}^{D}\;x_{i}^{2}\right)$	[−10,10]	0
F12	Styblinski-Tang	$0.5\sum_{i=1}^{D}\;\left(x_{i}^{4}-16x_{i}^{2}+5x_{i}\right)$	[−5,5]	−39.116 × *d*
F13	Rastrigin	$\sum_{i=1}^{D}\;\left[x_{i}^{2}-10\cos\left(2\pi x_{i}\right)+10\right]$	[−5.12,5.12]	0
F14	Ackey 1	$\begin{array}{c} -20\exp\;\left(-0.2\sqrt{\frac{\left(\sum_{i=1}^{D}\;x_{i}^{2}\right)}{D}}\right)- \end{array}\exp\;\left(\frac{\left(\sum_{i=1}^{D}\;\cos\left(2\pi x_{i}\right)\right)}{D}\right)$ + 20 + e	[−32,32]	0
F15	Griewank	$\frac{\sum_{i=1D}^{D}\;x_{i}^{2}}{4000}-\prod_{i=1}^{D}\;\cos\left(\frac{x_{i}}{\sqrt{i}}\right)+1$	[−600,600]	0
F16	Xin-She Yang N.4	$(\sum_{i=1}^{D}\;\sin^{2}\;\left(x_{i}\right)-\exp\;(-\sum_{i=1}^{D}\;x_{i}^{2}))exp\;(-\sum_{i=1}^{D}\;\sin^{2}\sqrt{\left|x_{i}\right|})$	[−10,10]	−1
F17	Penalized	$\frac{\pi }{D}\{10\sin\left(\pi y_{1}\right)+\sum_{i=1}^{D}\;{\left(y_{i}-1\right)}^{2}\left[1+10\sin^{2}\left(\pi y_{i+1}\right)\right]+{\left(y_{n}-1\right)}^{2}\}+\sum_{i=1}^{D}\;u\left(x_{i},10,100,4\right)$	[−50,50]	0
F18	Penalized2	$\begin{array}{c} 0.1\{\sin^{2}\left(3\pi x_{1}\right)+\sum_{i=1}^{D}\;{\left(x_{i}-1\right)}^{2}\left[1+\sin^{2}\left(3\pi x_{i}+1\right)\right]\\ +{\left(x_{D}-1\right)}^{2}\left[1+\sin^{2}\left(2\pi x_{D}\right)\right]\}+\sum_{i=1}^{D}\;u\left(x_{i},5,100,4\right) \end{array}$	[−50,50]	0
F19	Foxholes	${\left[\frac{1}{500}+\sum_{j=1}^{25}\;\frac{1}{j+\sum_{i=1}^{2}\;{\left(x_{i}-a_{ij}\right)}^{6}}\right]}^{-1}$	±65.536	0.998
F20	Kowalik	$\sum_{i=1}^{11}\;{\left|a_{i}-\frac{x_{1}\left(b_{i}^{2}+b_{i}x_{2}\right)}{b_{i}^{2}+b_{i}x_{3}+x_{4}}\right|}^{2}$	[−5,5]	0.000308
F21	Six Hump Camel	$4x_{1}^{2}-2.1x_{1}^{4}+\frac{x_{1}^{6}}{3}+x_{1}x_{2}-4x_{2}^{2}+4x_{2}^{4}$	[−5,5]	−1.0316
F22	Shekel 5	$-\sum_{i=1}^{5}\;{\left|\left(x_{i}-a_{i}\right){\left(x_{i}-a_{i}\right)}^{T}+c_{i}\right|}^{-1}$	[0,10]	−10.1532
F23	Shekel 7	$-\sum_{i=1}^{7}\;{\left|\left(x_{i}-a_{i}\right){\left(x_{i}-a_{i}\right)}^{T}+c_{i}\right|}^{-1}$	[0,10]	−10.4028
F24	Shekel 10	$-\sum_{i=1}^{10}\;{\left|\left(x_{i}-a_{i}\right){\left(x_{i}-a_{i}\right)}^{T}+c_{i}\right|}^{-1}$	[0,10]	−10.5364

**Table 3:** Details of benchmark problems for composition functions.

	Name	Function	Brief Expressions	$f_{min}$
F25	CEC2017-F21	Composition Function 1 (N = 3)	$\sum_{i=1}^{N}\;\omega_{i}\cdot \left(\lambda_{i}\cdot g_{i}(x)+{\;\mathrm{bias}\;}_{i}\right)+F_{21}^{*},g(x)=\left\{f_{4},f_{11},f_{5}\right\}$	2100
F26	CEC2017-F22	Composition Function 2 (N = 3)	$\sum_{i=1}^{N}\;\omega_{i}\cdot \left(\lambda_{i}\cdot g_{i}(x)+{\;\mathrm{bias}\;}_{i}\right)+F_{22}^{*},g(x)=\left\{f_{5},f_{15},f_{10}\right\}$	2200
F27	CEC2017-F23	Composition Function 3 (N = 4)	$\sum_{i=1}^{N}\;\omega_{i}\cdot \left(\lambda_{i}\cdot g_{i}(x)+{\;\mathrm{bias}\;}_{i}\right)+F_{23}^{*},g(x)=\left\{f_{4},f_{13},f_{10},f_{5}\right\}$	2300
F28	CEC2017-F24	Composition Function 4 (N = 4)	$\sum_{i=1}^{N}\;\omega_{i}\cdot \left(\lambda_{i}\cdot g_{i}(x)+{\;\mathrm{bias}\;}_{i}\right)+F_{24}^{*},g(x)=\left\{f_{13},f_{11},f_{15},f_{5}\right\}$	2400
F29	CEC2017-F25	Composition Function 5 (N = 5)	$\sum_{i=1}^{N}\;\omega_{i}\cdot \left(\lambda_{i}\cdot g_{i}(x)+{\;\mathrm{bias}\;}_{i}\right)+F_{25}^{*},g(x)=\left\{f_{5},f_{17},f_{13},f_{12},f_{4}\right\}$	2500
F30	CEC2017-F26	Composition Function 6 (N = 5)	$\sum_{i=1}^{N}\;\omega_{i}\cdot \left(\lambda_{i}\cdot g_{i}(x)+{\;\mathrm{bias}\;}_{i}\right)+F_{26}^{*},g(x)=\left\{f_{6},f_{10},f_{15},f_{4},f_{5}\right\}$	2600

**Table 5:** Various BWOs from three strategies.

	CCM	FA	CMT
BWO	0	0	0
CCM_BWO	1	0	0
CMT_BWO	0	0	1
FA_BWO	0	1	0
CCM_CMT_BWO	1	0	1
CCM_FA_BWO	1	1	0
CMT_FA_BWO	0	1	1
FAMBWO	1	1	1

**Table 6:** Friedman test results on benchmark functions (F1–F30, dim = 30).

	Rank	+/−/=	Avg
FAMBWO	1	~	1.4
BWO	7	22/1/7	5.2
CCM_BWO	8	23/0/7	5.2333
CMT_BWO	6	23/0/7	4.5333
FA_BWO	5	22/1/7	4.5333
CCM_CMT_BWO	3	20/3/7	2.9
CCM_FA_BWO	4	21/2/7	3.6333
CMT_FA_BWO	2	20/3/7	1.9333

**Table 8:** Friedman test results on multimodal functions (F10–F24, dim = 30).

Fun	Rank	Avg
FAMBWO	1	1.6666
PSO	9	8.0
MFO	10	8.2
DE	8	7.6667
SCA	12	9.4667
SSA	11	8.8
WOA	7	7.1333
SOA	4	4.2
GWO	6	6.2
DBO	5	5.8667
WSO	2	2.4
BWO	3	3.1333

**Table 9:** Friedman test results on composition functions (F25–F30, dim = 30).

Fun	Rank	Avg
FAMBWO	1	1.1667
PSO	8	6.5
MFO	5	6
DE	3	4
SCA	10	9.3333
SSA	7	6.3333
WOA	4	4.8333
SOA	12	11.1667
GWO	11	10.8333
DBO	6	6.1167
WSO	9	8.5
BWO	2	3.1667

**Table 10:** Friedman test results on high-dimensional composition functions (F25–F30, dim = 100).

Fun	Rank	Avg
FAMBWO	1	1.1667
PSO	8	9.1667
MFO	5	5.6667
DE	3	6.5
SCA	10	5.5
SSA	7	8.5
WOA	4	6.1667
SOA	12	5.3333
GWO	11	6.8333
DBO	6	9.1667
WSO	9	7
BWO	2	7

**Table 11:** Search space for the 4 hyperparameters in FAMBWO-MA-BiLSTM.

Hyperparameter	Description	Range
m	Units in the BiLSTM layer	(32~128)
r	The parameter of the dropout layer, representing the proportion of dropout	(0.1~0.4)
learning rate	Hyperparameter in model training that controls the step size of the model during each iteration of parameter updates	(0.05~0.2)
batch size	Hyperparameter in model training, representing the number of samples used in each iteration during training	(32~128)

**Table 12:** Comparison of prediction performance.

	MSE(TECU)	RMSE(TECU)	MAE(TECU)	$R^{2}$
GS-MA-BiLSTM	10.1761	3.19	2.28	0.9697
RS-MA-BiLSTM	9.7969	3.13	2.19	0.9718
BOA-MA-BiLSTM	9.4864	3.08	2.15	0.9725
BWO-MA-BiLSTM	8.8209	2.97	2.06	0.9782
FAMBWO-MA-BiLSTM	8.2944	2.88	1.97	0.9803

## Data Availability

The raw data supporting the conclusions of this article will be made available by the authors on request.

## Appendix A. Supplementary Section

**Table A1:** Experimental results of strategy comparison on benchmark functions (F1–F30, dim = 30).

	**F1**		**F2**		**F3**	
	**Aver**	**Std**	**Aver**	**Std**	**Aver**	**Std**
BWO	6.0326E-97	1.1981E-96	2.0445E-49	3.6330E-49	6.5026E-66	1.4008E-65
CCM_BWO	8.5037E-98	5.4601E-98	7.2947E-48	3.7601E-48	8.0273E-65	9.2481E-65
CMT_BWO	4.8204E-106	7.6152E-105	5.2637E-56	8.3460E-56	4.2386E-69	3.471E-69
FA_BWO	1.7365E-102	3.1267E-101	2.1898E-51	4.7816E-51	2.8723E-72	7.9173E-72
CCM_CMT_BWO	3.5805E-127	8.4561E-127	6.6735E-65	7.2378E-65	1.0287E-86	5.2654E-86
CCM_FA_BWO	6.6154E-102	4.1783E-101	3.3487E-51	8.2376E-51	6.2377E-73	7.0192E-73
CMT_FA_BWO	4.8341E-138	7.1623E-138	8.2836-69	1.1687-69	3.1276E-89	7.2703E-89
FAMBWO	2.1784E-144	6.5303E-144	2.6852E-73	5.8304E-73	5.2865E-89	1.5806E-88
	F4		F5		F6	
	Aver	Std	Aver	Std	Aver	Std
BWO	5.2629E-94	1.2195E-93	3.3304E-48	5.0039E-48	7.8603E-48	1.6408E-47
CCM_BWO	5.3237E-97	4.3234E-96	7.1761E-52	1.0473E-52	8.1026E-52	6.3190E-51
CMT_BWO	3.2031E-118	6.1250E-116	4.2308E-64	3.7832E-64	3.7657E-64	3.1236E-64
FA_BWO	1.8723E-87	4.2134E-86	6.2167E-42	9.7912E-41	4.2386E-42	2.6124E-41
CCM_CMT_BWO	2.9323E-125	1.2987E-125	8.1268E-68	5.9473E-68	6.3201E-68	1.2081E-67
CCM_FA_BWO	7.1730E-104	5.6245E-103	3.0923E-57	2.3893E-57	2.5712E-57	8.6121E-56
CMT_FA_BWO	8.7163E-129	7.8160E-129	5.4861E-70	4.4012E-69	1.1863E-70	5.6737E-69
FAMBWO	1.8723E-143	2.7901E-143	3.9239E-71	1.0511E-70	3.0652E-71	5.9035E-71
	F7		F8		F9	
	Aver	Std	Aver	Std	Aver	Std
BWO	1.7754E+0	5.5606E-1	2.1725E-4	1.0442E-4	4.6357E-99	6.7786E-99
CCM_BWO	1.5011E+0	7.3688E-1	2.3125E-4	1.2023E-4	8.3871E-106	5.0871E-105
CMT_BWO	8.3679E-1	5.1682E-1	7.0761E-3	8.3561E-3	6.0964E-126	1.2944E-126
FA_BWO	6.8671E-1	4.3061E-1	3.4123E-4	7.1098E-3	8.8713E-114	3.4103E-114
CCM_CMT_BWO	4.1264E-1	3.8735E-1	5.1276E-4	6.1263E-4	1.3760E-133	4.2301E-133
CCM_FA_BWO	5.3672E-1	8.3877E-1	9.9760E-4	4.3872E-4	9.1367E-139	8.0122E-138
CMT_FA_BWO	2.2378E-1	3.1265E-1	1.9937E-4	9.3012E-5	3.7650E-130	1.2312E-139
FAMBWO	1.0229E-1	4.0150E-2	1.8683E-4	8.1479E-5	9.2454E-149	2.1089E-148
	F10		F11		F12	
	Aver	Std	Aver	Std	Aver	Std
BWO	-8.4003E+3	1.7763E+3	0.0000E+4	0.0000E+4	-1.0891E+3	4.1222E+1
CCM_BWO	-8.2371E+3	1.8761E+3	0.0000E+4	0.0000E+4	-1.0880E+3	4.2613E+1
CMT_BWO	-9.0364E+3	7.7801E-1	0.0000E+4	0.0000E+4	-1.0923E+3	6.1876E+1
FA_BWO	-8.1875E+3	1.0715E+2	0.0000E+4	0.0000E+4	-1.0955E+3	2.1651E+1
CCM_CMT_BWO	-2.7611E+4	3.9120E+1	0.0000E+4	0.0000E+4	-1.1781E+3	7.3751E+0
CCM_FA_BWO	-3.7013E+4	5.8761E-1	0.0000E+4	0.0000E+4	-1.1211E+3	9.1256E+0
CMT_FA_BWO	-2.1762E+4	9.7178E-1	0.0000E+4	0.0000E+4	-1.0403E+3	6.82257E+0
FAMBWO	-1.2569E+4	3.5252E-2	0.0000E+4	0.0000E+4	-1.1687E+3	6.8210E+0
	F13		F14		F15	
	Aver	Std	Aver	Std	Aver	Std
BWO	0.0000E+4	0.0000E+4	4.4409E-16	0.0000E+4	0.0000E+4	0.0000E+4
CCM_BWO	0.0000E+4	0.0000E+4	4.4409E-16	0.0000E+4	0.0000E+4	0.0000E+4
CMT_BWO	0.0000E+4	0.0000E+4	4.4409E-16	0.0000E+4	0.0000E+4	0.0000E+4
FA_BWO	0.0000E+4	0.0000E+4	4.4409E-16	0.0000E+4	0.0000E+4	0.0000E+4
CCM_CMT_BWO	0.0000E+4	0.0000E+4	4.4409E-16	0.0000E+4	0.0000E+4	0.0000E+4
CCM_FA_BWO	0.0000E+4	0.0000E+4	4.4409E-16	0.0000E+4	0.0000E+4	0.0000E+4
CMT_FA_BWO	0.0000E+4	0.0000E+4	4.4409E-16	0.0000E+4	0.0000E+4	0.0000E+4
FAMBWO	0.0000E+4	0.0000E+4	4.4409E-16	0.0000E+4	0.0000E+4	0.0000E+4
	F16		F17		F18	
	Aver	Std	Aver	Std	Aver	Std
BWO	-1.0000E+0	0.0000E+4	1.1166E-1	3.8210E-2	1.5109E-3	1.3678E-3
CCM_BWO	-1.0000E+0	0.0000E+4	3.0762E-1	7.1267E-2	3.8730E-3	3.1287E-3
CMT_BWO	-1.0000E+0	0.0000E+4	6.7098E-1	1.2655E-2	5.1236E-3	2.8613E-3
FA_BWO	-1.0000E+0	0.0000E+4	7.7513E-2	4.6012E-2	1.1312E-3	2.0167E-3
CCM_CMT_BWO	-1.0000E+0	0.0000E+4	1.8075E-3	9.2606E-2	4.3760E-4	8.3106E-4
CCM_FA_BWO	-1.0000E+0	0.0000E+4	8.1338E-2	6.1763E-2	8.0983E-3	1.8971E-4
CMT_FA_BWO	-1.0000E+0	0.0000E+4	4.5362E-3	3.2780E-3	4.2371E-4	7.5608E-4
FAMBWO	-1.0000E+0	0.0000E+4	3.2652E-3	1.4095E-3	9.0821E-4	1.2501E-3
	F19		F20		F21	
	Aver	Std	Aver	Std	Aver	Std
BWO	9.9807E-1	1.3171E-4	4.6485E-4	5.2770E-4	-1.0316E+0	7.7597E-10
CCM_BWO	9.9807E-1	3.7072E-7	2.1901E-4	6.0981E-4	-1.0316E+0	6.8103E-10
CMT_BWO	9.9807E-1	7.9908E-4	5.1239E-4	9.8139E-4	-1.0316E+0	8.2385E-10
FA_BWO	9.9807E-1	2.8723E-10	9.1904E-4	1.2813E-4	-1.0316E+0	4.1324E-10
CCM_CMT_BWO	9.9807E-1	4.2831E-7	3.0736E-4	4.2874E-4	-1.0316E+0	7.1104E-10
CCM_FA_BWO	9.9807E-1	6.2903E-8	6.7938E-4	3.0982E-4	-1.0316E+0	5.9012E-10
CMT_FA_BWO	9.9807E-1	8.1308E-11	1.8341E-4	4.3703E-4	-1.0316E+0	6.1095E-10
FAMBWO	9.9807E-1	8.1593E-11	1.1780E-4	3.0644E-4	-1.0316E+0	5.9411E-10
	F22		F23		F24	
	Aver	Std	Aver	Std	Aver	Std
BWO	-8.0985E+0	1.8323E+0	-8.8582E+0	1.4560E+0	-8.5132E+0	1.3851E+0
CCM_BWO	-8.4208E+0	8.1983E-1	-3.4913E+0	4.3219E+0	-9.1278E+0	3.8902E-1
CMT_BWO	-9.0671E+0	1.6370E-1	-6.3098E+0	2.0344E-2	-9.7612E+0	4.7898E-1
FA_BWO	-6.7127E+0	7.6128E+0	-8.1298E+0	9.4089E+0	-1.0613E+1	8.3014E-2
CCM_CMT_BWO	-3.9820E+0	9.2891E+0	-1.4980E+1	3.4083E-1	-7.1975E+0	6.0781E-1
CCM_FA_BWO	-7.8087E+1	8.7087E-1	-9.8019E+0	4.3909E-1	-4.8035E+0	9.8776E-2
CMT_FA_BWO	-2.9083E+1	4.2389E-1	-4.8984E+0	5.9088E-1	-1.2049E+1	5.1780E-2
FAMBWO	-1.0119E+1	2.1270E-2	-1.0373E+1	2.5349E-2	-1.0489E+1	3.6462E-2
	F25		F26		F27	
	Aver	Std	Aver	Std	Aver	Std
BWO	4.0035E+3	4.0536E+2	5.9951E+4	8.5572E+3	3.7909E+4	4.3557E+3
CCM_BWO	4.0071E+3	4.9981E+2	5.9451E+4	6.7213E+3	3.8417E+4	4.6121E+3
CMT_BWO	4.0048E+3	5.9957E+2	5.8961E+4	7.7611E+3	3.7782E+4	3.6017E+3
FA_BWO	3.9983E+3	6.8903E+2	5.9071E+4	9.6713E+3	3.7076E+4	5.7104E+3
CCM_CMT_BWO	3.9008E+3	4.8482E+2	5.8601E+4	6.7613E+3	3.7892E+4	4.0913E+3
CCM_FA_BWO	3.9992E+3	3.8870E+2	5.8023E+4	7.6134E+3	3.6426E+4	6.8619E+3
CMT_FA_BWO	3.9601E+3	4.8611E+2	5.7761E+4	6.8731E+3	3.5716E+4	4.9125E+3
FAMBWO	3.8719E+3	3.2185E+2	5.6212E+4	6.3241E+3	3.3417E+4	3.8474E+3
	F28		F29		F30	
	Aver	Std	Aver	Std	Aver	Std
BWO	8.7766E+3	1.1405E+3	1.0724E+4	2.1617E+3	4.2551E+3	1.5691E+2
CCM_BWO	8.1880E+3	2.3927E+3	1.0246E+4	2.9831E+3	4.2671E+3	2.1013E+2
CMT_BWO	8.9967E+3	1.7853E+3	1.0497E+4	7.8087E+3	4.2401E+3	1.9954E+2
FA_BWO	8.9730E+3	1.1813E+3	1.0190E+4	4.1707E+3	4.1364E+3	2.0192E+2
CCM_CMT_BWO	8.0830E+3	2.8794E+3	9.9177E+3	2.5812E+3	4.2398E+3	1.8096E+2
CCM_FA_BWO	7.9884E+3	2.3971E+3	9.6019E+3	3.2991E+3	4.1043E+3	2.7913E+2
CMT_FA_BWO	7.9498E+3	1.8766E+3	9.0178E+3	4.8018E+3	4.1012E+3	1.0383E+2
FAMBWO	7.9028E+3	1.0457E+3	8.0399E+3	1.5892E+3	4.0977E+3	1.2075E+2

**Table A2:** Comparison results on unimodal functions (F1–F9, dim = 30).

Fun	Method	PSO	MFO	DE	SCA	SSA	WOA	SOA	GWO	DBO	WSO	BWO	FAMBWO
F1	Aver	6.8490E+0	6.7444E+3	3.4708E+4	2.2896E+2	5.0070E+3	4.0383E-7	1.4718E-83	1.8743E-11	1.0000E+3	2.4508E-13	6.0326E-97	2.1784E-144
	STD	1.7102E+0	3.4688E+3	4.1341E+3	2.0599E+2	3.0605E+3	1.1615E-6	2.6517E-83	1.3918E-11	3.0000E+3	5.1909E-13	1.1981E-96	6.5303E-144
F2	Aver	1.4574E+1	6.1632E+1	4.1621E+4	1.3758E+0	3.6748E+2	1.8221E-6	2.2915E-49	2.6769E-7	1.0000E+1	2.9476E-8	2.0445E-49	2.6852E-73
	STD	1.0815E+1	1.4123E+1	6.9389E+4	1.1781E+0	7.8082E+2	1.4165E-6	6.8722E-49	9.4134E-8	1.0000E+1	5.3270E-8	3.6330E-49	5.8304E-73
F3	Aver	3.2366E-2	6.8955E-3	7.7184E-1	1.1308E-2	1.4129E-2	7.7084E-26	4.5174E-75	8.2865E-36	4.8427E-21	1.2328E-18	6.5026E-66	5.2865E-89
	STD	3.2448E-2	4.7926E-3	2.5389E-1	2.3299E-2	1.0444E-2	1.3552E-74	1.3552E-74	1.1115E-35	1.1157E-20	2.1425E-18	1.4008E-65	1.5806E-88
F4	Aver	3.3500E+2	4.2556E+4	6.4232E+4	1.6679E+4	2.3164E+4	1.1590E+2	7.0551E+4	3.6043E-1	1.2141E+4	1.1838E-13	5.2629E-94	1.8723E-143
	STD	1.1339E+2	9.9362E+3	4.3655E+3	7.5642E+3	5.5044E+3	2.3903E+2	3.0165E+4	1.7346E-1	1.1663E+4	3.4892E-13	1.2195E-93	2.7901E-143
F5	Aver	3.9709E+0	7.4761E+1	8.6668E+1	4.8122E+1	5.0257E+1	2.0319E-1	2.2309E-12	6.9860E-3	1.2809E-9	6.3616E-8	3.3304E-48	3.9239E-71
	STD	1.2277E+0	6.1183E+0	2.7822E+0	7.3415E+0	5.4787E+0	1.9208E-1	5.6215E-12	3.7966E-3	1.9993E-9	1.7728E-7	5.0039E-48	1.0511E-70
F6	Aver	4.1819E+0	7.4350E+1	8.5470E+1	4.8395E+1	5.2017E+1	1.6344E-1	2.3028E-9	7.2349E-3	9.8127E-10	6.3104E-9	7.8603E-48	3.0652E-71
	STD	1.0668E+0	8.7207E+0	3.3904E+0	9.7793E+0	3.1908E+0	1.4722E-1	6.9018E-9	3.2801E-3	1.0469E-9	6.5178E-9	1.6408E-47	5.9035E-71
F7	Aver	7.3372E+0	6.7066E+3	3.5637E+4	9.1885E+2	2.2072E+3	1.2211E+0	1.2162E-1	6.5462E-1	1.0106E+3	4.1423E-2	1.7754E+0	1.0229E-1
	STD	2.8164E+0	2.9561E+3	7.0200E+3	8.4759E+2	1.0926E+3	4.2589E-1	1.3762E-1	3.1512E-1	3.0300E+3	3.7514E-2	5.5606E-1	4.0150E-2
F8	Aver	3.3204E+0	1.3878E+1	4.8039E+1	6.4379E-1	2.9030E+0	4.0584E-3	2.5258E-4	3.8932E-3	1.9174E-3	4.8033E-3	2.1725E-4	1.0442E-4
	STD	2.0680E+0	8.3744E+0	6.6963E+0	7.1711E-1	1.5040E+0	4.8341E-3	1.7940E-4	2.4039E-3	8.0869E-4	3.4241E-3	1.8683E-4	8.1479E-5
F9	Aver	1.0263E+2	2.3871E+2	2.3004E+2	6.3463E+0	2.2871E+1	3.5438E-7	1.1471E+0	2.9268E-12	1.0480E+1	9.9794E-14	4.6357E-99	9.2454E-149
	STD	1.7766E+2	6.8304E+1	4.1702E+1	3.8758E+0	1.1263E+1	3.5888E-7	3.4080E+0	3.0885E-12	3.1441E+1	2.9345E-13	6.7786E-99	2.1089E-148

**Table A3:** *p*-values of unimodal functions (F1–F9, dim = 30).

Fun	PSO	MFO	DE	SCA	SSA	WOA	SOA	GWO	DBO	WSO	BWO
F1	1.5705E-04	1.5705E-04	1.5705E-04	1.5705E-04	1.5705E-04	1.5705E-04	1.5705E-04	1.5705E-04	1.5705E-04	1.5705E-04	1.5705E-04
F2	1.5705E-04	1.5705E-04	1.5705E-04	1.5705E-04	1.5705E-04	1.5705E-04	1.5705E-04	1.5705E-04	1.5705E-04	1.5705E-04	1.5705E-04
F3	1.5705E-04	1.5705E-04	1.5705E-04	1.5705E-04	1.5705E-04	1.5705E-04	3.1971E-03	1.5705E-04	1.5705E-04	1.5705E-04	1.5705E-04
F4	1.5705E-04	1.5705E-04	1.5705E-04	1.5705E-04	1.5705E-04	1.5705E-04	1.5705E-04	1.5705E-04	1.5705E-04	1.5705E-04	1.5705E-04
F5	1.5705E-04	1.5705E-04	1.5705E-04	1.5705E-04	1.5705E-04	1.5705E-04	1.5705E-04	1.5705E-04	1.5705E-04	1.5705E-04	1.5705E-04
F6	1.5705E-04	1.5705E-04	1.5705E-04	1.5705E-04	1.5705E-04	1.5705E-04	1.5705E-04	1.5705E-04	1.5705E-04	1.5705E-04	1.5705E-04
F7	1.5705E-04	1.5705E-04	1.5705E-04	1.5705E-04	1.5705E-04	1.5705E-04	4.4969E-01	1.9397E-03	1.5705E-04	8.151E-03	1.5705E-04
F8	1.5705E-04	1.5705E-04	1.5705E-04	1.5705E-04	1.5705E-04	1.5705E-04	5.8782E-02	1.5705E-04	1.5705E-04	1.5705E-04	1.3057E-01
F9	1.5705E-04	1.5705E-04	1.5705E-04	1.5705E-04	1.5705E-04	1.5705E-04	1.5705E-04	1.5705E-04	1.5705E-04	1.5705E-04	1.5705E-04

**Table A4:** Comparison results on multimodal functions (F10–F24, dim = 30).

Fun	Method	PSO	MFO	DE	SCA	SSA	WOA	SOA	GWO	DBO	WSO	BWO	FAMBWO
F10	Aver	-4.1990E+3	-7.5453E+3	-5.3132E+3	-3.7296E+3	-6.9819E+3	-6.9421E+3	-1.2529E+4	-6.2970E+3	-9.5608E+3	-1.2568E+4	-8.4003E+3	-1.2569E+4
	STD	7.9674E+2	5.3018E+2	3.6783E+2	2.0742E+2	6.0326E+2	2.6357E+2	8.8831E+1	5.8555E+2	1.2744E+3	1.8889E+0	1.7763E+3	3.5252E-2
F11	Aver	6.1700E+0	4.3716E+0	7.0070E+0	7.5077E+0	2.3187E+0	8.2724E-1	0.0000E+4	1.4655E+0	3.7968E+0	1.2545E-10	0.0000E+4	0.0000E+4
	STD	8.6362E-1	4.5897E-1	3.1413E-1	5.8903E-1	6.8595E-1	5.5481E-1	0.0000E+4	3.3570E-1	1.3110E+0	2.2972E-10	0.0000E+4	0.0000E+4
F12	Aver	-8.6276E+2	-9.7358E+2	-5.3341E+2	-5.8953E+2	-9.1291E+2	-7.5878E+2	-1.1748E+3	-9.3520E+2	-8.1231E+2	-1.1749E+3	-1.0891E+3	-1.1687E+3
	STD	6.8209E+1	2.7682E+1	5.2359E+1	3.9380E+1	5.9464E+1	9.2879E+1	2.6498E-1	4.9462E+1	9.0867E+1	1.3952E-1	4.1222E+1	6.8210E+0
F13	Aver	1.9278E+2	2.1788E+2	3.5404E+2	6.5252E+1	2.1911E+2	1.0446E-1	0.0000E+4	1.5567E+1	5.7849E+0	1.2417E-13	0.0000E+4	0.0000E+4
	STD	2.4352E+1	4.2181E+1	1.5667E+1	2.8560E+1	3.7967E+1	3.1334E-1	0.0000E+4	5.9490E+0	1.1570E+1	3.4605E-13	0.0000E+4	0.0000E+4
F14	Aver	4.0415E+0	1.9160E+1	1.9959E+1	1.5741E+1	1.6377E+1	8.8903E-5	4.4409E-16	1.1499E-6	4.1993E-11	1.2470E-8	4.4409E-16	4.4409E-16
	STD	5.6701E-1	2.3175E+0	3.4900E-3	6.8663E+0	2.7163E+0	5.7951E-5	0.0000E+4	3.9160E-7	5.3902E-11	2.0079E-8	0.0000E+4	0.0000E+4
F15	Aver	8.6986E-1	6.5211E+1	3.2148E+2	3.2698E+0	2.8432E+1	6.9398E-3	0.0000E+4	6.3183E-3	9.1704E-15	7.5551E-14	0.0000E+4	0.0000E+4
	STD	5.4028E-2	4.2311E+1	6.6860E+1	2.4180E+0	1.0714E+1	1.1575E-2	0.0000E+4	7.9812E-3	2.7511E-14	1.9832E-13	0.0000E+4	0.0000E+4
F16	Aver	9.0278E-12	2.3995E-11	3.3364E-9	3.5848E-10	2.7083E-11	3.1951E-12	-1.0000E+0	6.1424E-15	5.1416E-12	-9.9876E-1	-1.0000E+0	-1.0000E+0
	STD	1.5150E-11	2.2714E-11	1.8871E-9	1.8679E-10	4.2416E-11	1.7624E-12	0.0000E+4	8.9887E-15	2.4031E-12	3.4803E-3	0.0000E+4	0.0000E+4
F17	Aver	1.9888E+0	7.1808E+6	2.5231E+8	6.5496E+6	1.5142E+6	1.3017E-1	1.8572E-2	6.2936E-2	3.8577E-2	3.5579E-3	1.1166E-1	3.2652E-3
	STD	8.0797E-1	6.1358E+6	1.1172E+8	1.4997E+7	1.8196E+6	1.5138E-1	8.6800E-3	4.6080E-2	1.4023E-2	6.9370E-4	3.8210E-2	1.4095E-3
F18	Aver	1.4771E+0	7.5675E+2	1.1689E+3	1.0721E+1	4.9256E+2	1.1190E+0	5.7996E-2	3.5672E-1	3.5387E-1	1.3311E-2	1.5109E-3	1.3678E-3
	STD	5.2995E-1	3.0941E+2	1.7358E+2	3.8644E+0	1.7474E+2	2.0245E-1	3.6602E-2	1.6280E-1	1.1955E-1	2.7532E-2	9.0821E-4	1.2501E-3
F19	Aver	2.0867E+0	1.3952E+0	9.9800E-1	1.5957E+0	2.1257E+0	1.4478E+0	3.3479E+0	3.7370E+0	1.0974E+0	9.9800E-1	9.9800E-1	9.9800E-1
	STD	1.4936E+0	6.5841E-1	1.2162E-16	9.0764E-1	1.2421E+0	6.6106E-1	3.7865E+0	4.1036E+0	2.9821E-1	1.7189E-12	1.3171E-4	8.1593E-11
F20	Aver	1.4243E-3	1.1496E-3	5.1773E-4	1.1113E-3	4.5175E-3	4.6798E-4	1.5745E-3	4.4585E-3	3.4553E-3	4.1358E-4	4.6485E-4	4.2770E-4
	STD	6.8454E-4	3.1737E-4	3.5571E-4	4.2714E-4	4.1546E-3	2.7070E-4	1.1457E-3	7.9532E-3	3.2167E-3	2.7329E-4	1.1780E-4	3.0644E-4
F21	Aver	-1.0315E+0	-1.0316E+0	-1.0316E+0	-1.0316E+0	-1.0316E+0	-1.0316E+0	-1.0316E+0	-1.0316E+0	-1.0316E+0	-1.0316E+0	-1.0316E+0	-1.0316E+0
	STD	1.5763E-4	1.4043E-16	7.8918E-15	8.0073E-5	1.2274E-13	4.8436E-8	4.0879E-6	4.8763E-8	6.2923E-8	3.7059E-14	7.7597E-10	5.9411E-10
F22	Aver	-1.0046E+1	-5.6140E+0	-1.0153E+1	-2.9240E+0	-5.4023E+0	-3.7643E+0	-8.8029E+0	-9.6446E+0	-7.0929E+0	-1.0153E+1	-8.0985E+0	-1.0119E+1
	STD	1.3707E-1	2.4519E+0	1.6085E-6	1.3862E+0	3.2428E+0	1.9736E+0	2.4198E+0	1.5158E+0	2.4738E+0	1.5392E-5	1.8323E+0	2.1270E-2
F23	Aver	-1.0285E+1	-7.6342E+0	-1.0403E+1	-3.4061E+0	-5.7208E+0	-3.9907E+0	-7.0898E+0	-9.8684E+0	-9.3005E+0	-1.0403E+1	-8.8582E+0	-1.0373E+1
	STD	7.5974E-2	3.4014E+0	7.9317E-6	1.2397E+0	3.1696E+0	1.4778E+0	2.9727E+0	1.5936E+0	2.0919E+0	8.8006E-6	1.4560E+0	2.5349E-2
F24	Aver	-1.0450E+1	-5.3728E+0	-1.0536E+1	-3.5850E+0	-6.3097E+0	-4.0946E+0	-5.0826E+0	-1.0534E+1	-7.7791E+0	-1.0536E+1	-8.5132E+0	-1.0489E+1
	STD	9.2939E-2	2.7765E+0	3.1115E-6	1.3324E+0	3.5236E+0	1.8502E+0	2.7384E+0	1.3937E-3	2.7125E+0	2.0858E-5	1.3851E+0	3.6462E-2

**Table A5:** *p*-values of multimodal functions (F10–F24, dim =30).

Fun	PSO	MFO	DE	SCA	SSA	WOA	SOA	GWO	DBO	WSO	BWO
F10	1.5705E-04	1.5705E-04	1.5705E-04	1.5705E-04	1.5705E-04	1.5705E-04	4.4969E-01	1.5705E-04	2.4969E-03	1.5093E-01	1.5705E-04
F11	1.5705E-04	1.5705E-04	1.5705E-04	1.5705E-04	1.5705E-04	1.5705E-04	1E+00	1.5705E-04	1.5705E-04	1.5705E-04	1E+00
F12	1.5705E-04	1.5705E-04	1.5705E-04	1.5705E-04	1.5705E-04	1.5705E-04	1.5705E-04	1.5705E-04	1.5705E-04	1.5705E-04	1.5705E-04
F13	1.5705E-04	1.5705E-04	1.5705E-04	1.5705E-04	1.5705E-04	1.5705E-04	1E+00	1.5705E-04	5.8782E-02	5.8782E-02	1E+00
F14	1.5705E-04	1.5705E-04	1.5705E-04	1.5705E-04	1.5705E-04	1.5705E-04	1E+00	1.5705E-04	1.5705E-04	1.5705E-04	1E+00
F15	1.5705E-04	1.5705E-04	1.5705E-04	1.5705E-04	1.5705E-04	1.5705E-04	1E+00	1.5705E-04	7.0546E-01	1.3057E-01	1E+00
F16	1.5705E-04	1.5705E-04	1.5705E-04	1.5705E-04	1.5705E-04	1.5705E-04	1E+00	1.5705E-04	1.5705E-04	1.5705E-04	1E+00
F17	1.5705E-04	1.5705E-04	1.5705E-04	1.5705E-04	1.5705E-04	1.5705E-04	1.5705E-04	1.5705E-04	1.5705E-04	1.9876E-01	1.5705E-04
F18	1.5705E-04	1.5705E-04	1.5705E-04	1.5705E-04	1.5705E-04	1.5705E-04	1.5705E-04	1.5705E-04	1.5705E-04	4.072E-03	6.5015E-01
F19	8.2099E-02	4.4969E-01	1.5705E-04	1.5705E-04	2.3342E-02	1.5705E-04	1.7362E-01	3.8106E-04	4.4969E-01	3.8106E-04	1.5705E-04
F20	1.4989E-03	6.5017E-03	2.2648E-01	8.151E-03	3.8106E-04	4.0568E-01	4.9366E-02	2.8992E-01	8.8074E-04	1.0165E-02	5.967E-01
F21	1.5705E-04	1.5705E-04	1.5705E-04	1.5705E-04	1.5705E-04	3.8106E-04	1.5705E-04	1.5705E-04	1.5705E-04	1.5705E-04	2.2648E-01
F22	5.8782E-02	2.3342E-02	1.5705E-04	1.5705E-04	1.3057E-01	1.5705E-04	1.5093E-01	1.0165E-02	3.4294E-02	1.5705E-04	1.5705E-04
F23	1.152E-03	4.4969E-01	1.5705E-04	1.5705E-04	1.3057E-01	1.5705E-04	2.4969E-03	2.4969E-03	4.9366E-02	1.5705E-04	1.5705E-04
F24	7.0546E-01	2.3342E-02	1.5705E-04	1.5705E-04	4.4969E-01	1.5705E-04	2.1218E-04	1.5705E-04	1.3057E-01	1.5705E-04	2.1218E-04

**Table A6:** Comparison results on composition functions (F25–F30, dim = 30).

Fun	Method	PSO	MFO	DE	SCA	SSA	WOA	SOA	GWO	DBO	WSO	BWO	FAMBWO
F25	Aver	4.2501E+3	4.3714E+3	4.2012E+3	4.1697E+3	4.2810E+3	4.1909E+3	4.2513E+3	4.1745E+3	4.1032E+3	4.3108E+3	4.0035E+3	3.8719E+3
	STD	3.9414E+2	4.1368E+2	3.9646E+2	5.5081E+2	4.4508E+2	4.7587E+2	4.1419E+2	3.2691E+2	4.3694E+2	4.8812E+2	4.0536E+2	3.2185E+2
F26	Aver	6.1497E+4	6.2600E+4	6.3064E+4	6.1810E+4	6.3679E+4	6.2453E+4	6.2085E+4	6.3440E+4	6.4170E+4	6.3717E+4	5.9951E+4	5.6212E+4
	STD	8.3180E+3	6.1200E+3	7.9024E+3	5.9720E+3	6.7052E+3	7.1979E+3	5.2581E+3	7.8692E+3	5.9357E+3	6.0638E+3	8.5572E+3	6.3241E+3
F27	Aver	3.7884E+4	3.7348E+4	3.7175E+4	3.7916E+4	3.8343E+4	3.8939E+4	4.0013E+4	3.8708E+4	3.7545E+4	3.9364E+4	3.7909E+4	3.3417E+4
	STD	4.8197E+3	4.8688E+3	5.3916E+3	4.4953E+3	3.8023E+3	3.7373E+3	4.5948E+3	3.8914E+3	4.0606E+3	4.4915E+3	4.3557E+3	3.8474E+3
F28	Aver	9.2863E+3	9.0139E+3	9.1491E+3	9.1018E+3	9.1074E+3	8.9788E+3	8.9313E+3	9.0204E+3	9.0009E+3	9.1428E+3	8.7766E+3	7.9028E+3
	STD	1.0664E+3	1.1657E+3	1.3429E+3	1.5160E+3	1.2147E+3	1.1470E+3	1.3252E+3	1.2400E+3	9.8165E+2	1.3555E+3	1.1405E+3	1.0457E+3
F29	Aver	1.0741E+4	1.1438E+4	1.0546E+4	1.1140E+4	1.1237E+4	1.0864E+4	1.1093E+4	1.1180E+4	1.0915E+4	1.0611E+4	1.0724E+4	8.0399E+3
	STD	1.2948E+3	1.9296E+3	1.6449E+3	1.4688E+3	1.4112E+3	1.8017E+3	1.8848E+3	1.5839E+3	1.8441E+3	1.8707E+3	2.1617E+3	1.5892E+3
F30	Aver	4.2832E+3	4.3256E+3	4.3162E+3	4.2181E+3	4.2981E+3	4.2168E+3	4.2989E+3	4.3055E+3	4.2065E+3	4.2300E+3	4.2551E+3	4.0977E+3
	STD	1.3042E+2	2.2106E+2	1.5269E+2	2.4449E+2	2.1250E+2	1.5210E+2	1.5466E+2	1.6424E+2	1.6123E+2	1.1989E+2	1.5691E+2	1.2075E+2

**Table A7:** *p*-values of composition functions (F25–F30, dim = 30).

Fun	Dim	PSO	MFO	DE	SCA	SSA	WOA	SOA	GWO	DBO	WSO	BWO
F25	30	4.8461E-4	1.3825E-5	1.2046E-3	1.9494E-2	4.1013E-4	4.9689E-3	7.9060E-4	1.4796E-3	1.9494E-2	1.7317E-4	1.8332E-1
F26	30	1.7227E-3	3.0928E-4	1.5382E-4	1.1436E-3	7.8991E-5	1.2685E-3	3.8778E-4	5.4107E-4	2.3542E-5	4.7885E-5	5.9613E-3
F27	30	2.6046E-4	2.4390E-3	3.2599E-3	3.0928E-4	4.7885E-5	3.7006E-6	2.2340E-6	1.2077E-5	4.1013E-4	5.6570E-6	7.8991E-5
F28	30	3.2611E-5	8.3393E-4	9.2727E-4	7.4937E-4	8.3393E-4	7.4937E-4	4.1284E-3	1.8368E-4	3.8778E-4	1.0302E-3	2.5611E-3
F29	30	4.4937E-4	3.0201E-4	2.9436E-3	2.2988E-4	1.3065E-4	8.4421E-4	9.5315E-4	1.5084E-4	1.2093E-3	7.7931E-3	3.6203E-3
F30	30	1.5084E-3	1.5084E-3	1.7420E-4	1.0134E-1	6.5914E-3	1.3004E-1	1.5084E-3	2.6370E-3	4.0057E-2	1.2093E-2	7.4655E-3

**Table A8:** Comparison results on high-dimensional composition functions (F25–F30, dim = 100).

Fun	Dim	Method	PSO	MFO	DE	SCA	SSA	WOA	SOA	GWO	DBO	WSO	BWO	FAMBWO
F25	100	Aver	1.0385E+4	9.7473E+3	1.0265E+4	1.0293E+4	1.0963E+4	1.0336E+4	1.0300E+4	1.0696E+4	1.0301E+4	1.0099E+4	1.0465E+4	9.8810E+3
		STD	1.3243E+3	1.2451E+3	9.1069E+2	6.4140E+2	1.2945E+3	1.1711E+3	7.4290E+2	8.3234E+2	1.2352E+3	5.9307E+2	1.4310E+3	7.6980E+2
F26	100	Aver	1.4816E+5	1.4716E+5	1.4950E+5	1.4126E+5	1.4230E+5	1.4274E+5	1.4112E+5	1.4312E+5	1.4567E+5	1.4665E+5	1.4570E+5	1.3482E+5
		STD	7.3497E+3	6.5706E+3	1.0969E+4	1.8194E+4	1.0396E+4	8.3374E+3	1.2159E+4	9.3927E+3	1.3538E+4	1.3796E+4	8.5761E+3	1.0697E+4
F27	100	Aver	9.3549E+4	8.8460E+4	9.1208E+4	8.7080E+4	9.4635E+4	9.5866E+4	8.9455E+4	8.7414E+4	9.0502E+4	9.3059E+4	8.9737E+4	8.2718E+4
		STD	6.4928E+3	8.8652E+3	6.4811E+3	7.4193E+3	7.0810E+3	5.2458E+3	6.6586E+3	1.1432E+4	8.6436E+3	8.0090E+3	8.0301E+3	6.9324E+3
F28	100	Aver	3.5044E+4	3.5828E+4	3.4288E+4	3.4423E+4	3.5320E+4	3.4143E+4	3.4184E+4	3.4150E+4	3.5882E+4	3.5157E+4	3.5221E+4	3.0264E+4
		STD	4.4720E+3	3.2380E+3	3.4731E+3	4.1880E+3	3.0991E+3	3.9773E+3	3.3118E+3	4.8475E+3	3.8251E+3	4.2116E+3	4.8540E+3	3.2352E+3
F29	100	Aver	4.2600E+4	3.8342E+4	4.0075E+4	3.9187E+4	4.0305E+4	4.0275E+4	3.9146E+4	4.2023E+4	4.3092E+4	4.2620E+4	3.7676E+4	3.4992E+4
		STD	3.9281E+3	5.9466E+3	8.1387E+3	7.0578E+3	8.8507E+3	8.2231E+3	7.5353E+3	6.1876E+3	6.8596E+3	5.1902E+3	5.5419E+3	4.0076E+3
F30	100	Aver	5.8614E+3	5.7999E+3	5.7811E+3	5.9534E+3	5.8223E+3	5.7778E+3	5.9028E+3	5.8829E+3	5.9016E+3	5.7467E+3	5.8418E+3	5.5284E+3
		STD	3.1410E+2	3.0393E+2	2.8520E+2	1.7280E+2	3.5349E+2	4.4726E+2	1.4622E+2	3.6817E+2	3.1756E+2	2.3156E+2	2.8487E+2	2.0158E+2

**Table A9:** *p*-values of high-dimensional composition functions (F25–F30, dim = 100).

Fun	PSO	MFO	DE	SCA	SSA	WOA	SOA	GWO	DBO	WSO	BWO
F25	4.0057E-2	9.8345E-3	2.3716E-2	3.0953E-3	5.1139E-3	1.6468E-3	1.4089E-3	1.2093E-3	5.6468E-2	6.0413E-2	3.6203E-3
F26	1.1298E-3	3.0201E-3	3.0201E-3	4.0057E-2	9.2984E-2	5.9126E-2	1.0134E-1	7.7931E-2	2.3787E-2	2.1333E-2	1.0744E-2
F27	2.8416E-4	3.2670E-2	3.9425E-3	1.0134E-2	1.7420E-4	3.0655E-5	1.2093E-2	5.3764E-2	6.5914E-3	1.7386E-3	6.5914E-3
F28	5.8104E-3	2.8416E-4	5.8104E-3	4.4937E-3	9.7547E-4	1.3589E-2	5.1139E-3	4.4253E-2	1.1298E-3	3.0201E-3	6.5914E-3
F29	7.4588E-5	1.9136E-2	3.2670E-2	4.4253E-2	2.3787E-2	1.1984E-2	2.2110E-2	1.5084E-3	1.5084E-3	2.4178E-4	1.5243E-2
F30	2.6370E-3	8.4421E-3	1.5247E-2	3.6742E-5	1.9103E-2	9.5315E-3	7.4588E-5	5.1139E-3	9.7547E-4	1.9103E-2	6.5914E-3
